# Bioactive Composition of Tropical Flowers and Their Antioxidant and Antimicrobial Properties

**DOI:** 10.3390/foods13233766

**Published:** 2024-11-24

**Authors:** Elena Coyago-Cruz, Alejandro Barrigas, Aida Guachamin, Jorge Heredia-Moya, Johana Zuñiga-Miranda, Edwin Vera

**Affiliations:** 1 Carrera de Ingeniería en Biotecnología de los Recursos Naturales, Universidad Politécnica Salesiana, Sede Quito, Campus El Girón, Av. 12 de Octubre N2422 y Wilson, Quito 170109, Ecuador; 2 Maestría en Productos Farmacéuticos Naturales, Universidad Politécnica Salesiana, Sede Quito, Campus El Girón, Av. 12 de Octubre N2422 y Wilson, Quito 170109, Ecuador; 3 Centro de Investigación Biomédica (CENBIO), Facultad de Ciencias de la Salud Eugenio Espejo, Universidad UTE, Quito 170527, Ecuador; 4 Departamento de Ciencia de los Alimentos y Biotecnología, Facultad de Ingeniería Química, Escuela Politécnica Nacional, Quito 170524, Ecuador

**Keywords:** carotenoids, phenolics, organic acids, anthocyanins, micro-extraction, experimental design, PCA

## Abstract

This study evaluated tropical flower petals’ bioactive compounds and antioxidant and antimicrobial properties. The physicochemical characteristics, carotenoids, phenolics, anthocyanins, organic acids, and antioxidant activity of 67 flowers were analyzed. In addition, the antimicrobial activity against *Escherichia coli, Staphylococcus aureus, Pseudomonas aeruginosa*, *Streptococcus mutans, Candida albicans*, and *Candida tropicalis* of 35 species was determined. A 2 × 3 experimental design was used for the extraction of carotenoids and phenolics, including solvents and ultrasonic agitation times. The mixture of methanol–acetone–dichloromethane (1:1:2) and acetone–methanol (2:1) resulted in the highest concentration of carotenoids, while acidified 80% methanol favoured phenolic extraction. *Renealmia alpinia* was extremely rich in carotenoids (292.5 mg β-carotene/g DW), *Pleroma heteromallum* in anthocyanins (7.35 mg C-3-gl/g DW), while a high content of citric acid was found in *Hibiscus rosa-sinensis* (17,819 mg/100 g DW). On the other hand, *Thibaudia floribunda* showed the highest antioxidant activity (7.8 mmol Trolox equivalent/g DW). The main phenolics were *m*-coumaric acid in *Acalypha poiretii* (12,044 mg/100 g DW), 4-hydroxybenzoic acid in *Brugmansia arborea* (10,729 mg/100 g DW), and kaempferol in *Dahlia pinnata* (8236 mg/100 g DW). The extract of *Acalypha poiretii*, *Brownea macrophylla*, and *Cavendishia nobilis* showed antibacterial activity, while the extract of *Pleroma heteromallum* was the only one active against *Candida albicans*. These findings highlight the potential health benefits from certain tropical flowers.

## 1. Introduction

Tropical forests are characterized by average temperatures of 20–25 °C, abundant sunlight, and regular rainfall. These ecosystems cover 6% of the Earth’s land surface and are home to various species, including plants that provide food and medicine for local communities [1,2,3].

Within the vast diversity of plants, the flowers of several tropical species stand out for their content of bioactive compounds such as carotenoids, phenolic compounds, monophenols, and terpenes, which provide functional benefits beyond basic nutrition [4,5]. These compounds act as antioxidants and contribute to well-being through anti-inflammatory, immunomodulatory, anticarcinogenic, and cardiovascular protective activities. In addition, some of the carotenoids found in these plants can be converted into vitamin A, essential for eye health and the immune system, reinforcing the importance of these natural sources in the human diet [5,6,7,8,9]. Recent research has shown that the synergistic effect of bioactive compounds is more effective than the consumption of individual molecules. For example, eating foods high in carotenoids and phenolics is beneficial for cardiovascular disease, neurodegenerative disease, and certain types of cancer [4,10,11,12,13].

Families such as Asteraceae, Lamiaceae, Fabaceae, Apiaceae, Araliaceae, Ericaceae, Zingiberaceae, and others have been identified as valuable sources for the treatment of liver disease due to the presence of bioactive compounds in their floral structures. For example, the Asteraceae family, which contains more than 2500 flowering species, is important in traditional medicine and dietary practises. Similarly, the Lamiaceae family contains more than 7000 species, many of which are used for their essential oil content and medicinal applications [7,12,14].

On the other hand, extracting bioactive compounds for food and medicinal purposes is a significant challenge. Green chemistry methods using ultrasound, microwave-assisted extraction, and enzymatic extraction have significantly reduced solvent consumption and processing time. In particular, carotenoids require organic solvents such as hexane, methanol, acetone, petroleum ether, etc. For phenolic compounds, ethanol, methanol, water, and acetone are generally used, depending on the polarity of the target phenolics [15,16]. Another parameter to consider in this context is the extraction time, which is also critical, as a more extended time may result in a complete extraction but must be balanced with the efficiency of the process [17].

As the concentration of carotenoids and phenolics varies according to species, maturity, and growth conditions, it is essential to adapt extraction methods to maximize the yield of each compound [9]. In this context, this study focused on the evaluation of bioactive compounds, antioxidants, and antimicrobials in tropical flowers, using different assays to identify species with a high content of bioactive compounds that can be used in the food and pharmaceutical industries and contribute to the development of functional foods.

## 2. Materials and Methods

### 2.1. Reagents and Standards

The chemicals used in this investigation included acetone (CAS 67-64-1) and fluconazole (86386-73-4), which were reagent grade. At the same time, HPLC grade chemicals included acetonitrile (CAS 75-05-8), ethanol (CAS 64-17-5), and methanol (CAS 67-56-1), which were purchased from Fisher Chemical (Fischer Scientific Inc., Madrid, Spain). ABTS (2,2′-azino-bis-(3-ethylbenzothiazoline-6-sulfonic acid) (CAS 30931-67-0), DPPH (2,2-Diphenyl-1-picrylhydrazyl) (CAS 1898-66-4), formic acid (CAS 64-18-6), Folin–Ciocalteu (CAS 7732-18-5), metaphosphoric acid (CAS 37267-86-0), potassium chloride (CAS 7447-40.7), potassium persulphate (CAS 7727-21-1), sodium acetate try hydrate (CAS 6131-90.4), sodium carbonate (CAS 497-19-8), and sodium hydroxide (CAS 1310-73-2) were also used; all analytical grades were purchased from Sigma (Merck, Darmstadt, Germany). Hydrochloric acid (CAS 7647-01-0) was also obtained as an analytical grade from Labscan (RCI Labscan group, Dublin, Republic of Ireland). Brain Heart Infusion (BHI), Mueller Hinton agar (MHA), and Sabouraud dextrose agar (SDA) were purchased from BD DifcoTM (Fisher Scientific Inc., Madrid, Spain). Yeast Peptone Dextrose Broth (YPDB) from SRL (Sisco Research Laboratories Pvt. Ltd., Mumbai, India) and streptomycin sulphate sulphate (CAS 3810-74-0) from Phytotech (PhytoTechnology Laboratories ^®^, Lenexa, KS, USA). Water was purified using a NANOpureDiamondTM system (Barnsted Inc., Dubuque, IO, USA).

Standards include β-carotene 93.0% (CAS 7235-40-7), caffeic acid 98.0% (CAS 331-39-5), chlorogenic acid 95.0% (CAS 327-97-9), chrysin 97.0% (CAS 480-40-0), cyanidin-e-glucoside chloride 97.0% (CAS 7084-24-4), ferulic acid 100.0% (CAS 1135-24-6), gallic acid 100.0% (CAS 149-91-7), 2,5-dihydroxybenzoic acid 98.0% (CAS 490-79-9), 3-hydroxybenzoic acid 99.0% (CAS 99-06-3), kaempferol 97.0% (CAS 520-18-3), luteolin 98% (CAS 491-70-3), *m*-coumaric acid 99.0% (CAS 588-30-7), naringin 95.0% (CAS 10236-47-2), *o*-coumaric acid 97.0% (CAS 614-60-8), *p*-coumaric acid 98.0% (CAS 501-98-4), *p*-hydroxybenzoic acid 99,0% (CAS 99-06-3), quercetin 95.0% (CAS 849061-97-8), rutin 94.0% (CAS 153-18-4), shikimic acid 99.0% (CAS 138-59-0), syringic acid 95,0% (CAS 530-57-4), vanillic acid 97.0% (CAS 121-34-6), and Trolox 98% (CAS 53188-07-1), which were purchased from Sigma (Merck, Darmstadt, Germany). *Candida albicans* ATCC 1031, *Candida tropicalis* ATCC 13803, *Escherichia coli* ATCC 8739, *Pseudomonas aeruginosa* ATCC 9027, *Staphylococcus aureus* ATCC 6538P and, *Streptococcus mutans* ATCC 25175 were purchased from ATTC (ATTC, Manassas, VA, USA).

### 2.2. Physicochemical Quantification

**Figure 1:**
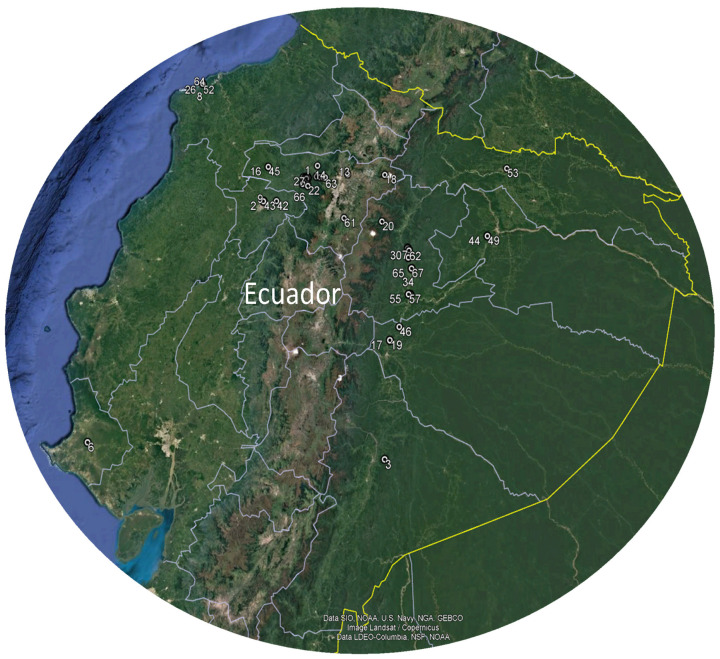
Geographical distribution of the flowers under study. Note: The numbers correspond to the number of blossoms examined (Table 1).

For this study, 67 different flower species from tropical areas of Ecuador were selected (Table 1 and Figure 1) and collected throughout the year 2020. Thirty flowers were randomly sampled from each species and considered for physicochemical analysis. These were placed in containers provided with floral foam to prevent deterioration. At the same time, for other studies, approximately 200 g of petals were selected and carefully stored in Falcon tubes and subjected to lyophilisation using the Christ Alpha 1-4 LDplus apparatus (Martin Gefriertrocknungsanlagen GmbH, Osterode am Harz, Germany). In addition, fresh samples of flowers, leaves, stems, and fruits were collected, pressed, and dried for botanical identification in QUPS-Ecuador Herbarium. The physicochemical analysis included the determination of weight, equatorial and longitudinal diameters, soluble solids, pH, total titratable acid, moisture., and ash following the protocol of Coyago et al. [9]. 

### 2.3. Optimization of Extraction Parameters and Quantification of Carotenoids

Micro-extractions were carried out in the dark and in triplicate. A 2 × 3 experimental design was implemented with solvent type and ultrasonic extraction time. Different solvent combinations, including acetone–methanol (2:1), *n*-hexane–acetone (1:1) and methanol–acetone–dichloromethane (1:1:2), were considered. In addition, the ultrasonic extraction time varied between 1, 2, and 3 min. Only the petals of *Taraxacum officinale*, *Pyrostegia venusta*, and *Buddleja globosa*, species known for their yellow colour and carotenoid content [18,19], were selected to optimize the ultrasonic extraction time and extraction solvent for the 67 samples. For the extraction of carotenoids according to the specific combinations of the experimental design, a mixture of 20 mg of lyophilised powder with one mL of the chosen solvent. This solution was then vortexed using a VM-300 vortex (Interbiolab Inc., Orlando, FL, USA) and shaken in a Fisher Scientific FS60 ultrasonicator (Fisher Scientific, Waltham, MA, USA) for the time specified in the experimental design. Subsequently, the mixture was centrifuged for 3 min at 1400 rpm and 4 °C using a MiniSpin microcentrifuge (Eppendorf, Bochum, Germany). The coloured phase was collected, and the extraction process was repeated until the solid residue became colourless. The coloured phase evaporated to dryness (below 30 °C) using a Buchi TM R-100 (Fisher Scientific, Waltham, MA, USA).

To quantify carotenoids by spectrophotometry, the dried extract was dissolved in 2 mL of ethanol and transferred to a 10 mm light path quartz cell. The absorbance of the solution was measured at 450 nm using a ThermoSpectromic Genesys 10 UV-Vis spectrophotometer (ThermoFisher Scientific, Waltham, MA, USA). A calibration curve was prepared using a 5 mg β-carotene standard dissolved in 25 mL of ethanol to ensure accurate measurements. The concentration of total carotenoids in the samples was expressed as micrograms of β-carotene per gram of freeze-dried petal weight (DW) (µg β-carotene/g DW) [20]. Finally, following the above procedure, the remaining samples were extracted using the solvent and for a time that maximized extraction.

### 2.4. Optimization of Extraction Parameters Andquantification of Phenolic Compounds

Micro-extraction was performed in triplicate. A 3 × 2 experimental design was implemented with solvent type and ultrasonic extraction time. Different aqueous solvents, including ethanol (75%), methanol (75%), and methanol (80%) acidified with hydrochloric acid (0.1%), were evaluated to determine the most efficient mixture for phenol extraction. In addition, the ultrasonic extraction time varied between 1, 3, and 5 min. Only the petals of *Dahlia pinnata, Dianthus caryophyllus, Plerona urvilleanum, Agapanthus africanus*, and *Lupinus mutabilis*, known for their red to purple colours and phenolic content [18,19] were selected to optimize the ultrasonic extraction time and extraction solvent for the 67 samples. To extract phenolics according to the specific combinations of the experimental design, 40 mg of lyophilised powder was mixed with 1 mL of the chosen solvent. This solution was then vortexed using a VM-300 vortex mixer (Interbiolab Inc., Orlando, FL, USA) and subjected to ultrasound in a Fisher Scientific FS60 (Fisher Scientific, Waltham, MA, USA) for the time specified in the experimental design. The supernatant was recovered by centrifugation at 1400 rpm for 5 min at 4 °C. The extraction was repeated twice using 500 µL of the methanolic solution. To remove impurities, the resulting methanolic extract was filtered through a 0.45 µm PVDF filter [5,21].

To quantify total phenolics, 20 µL of the methanolic extract was mixed with 100 µL of a 1:4 Folin–Cioacalteu solution and 75 µL of a sodium carbonate solution (100 g/L) in a 96-well plate. The mixture was homogenized and allowed to stand for two hours at room temperature. Absorbance was measured at 750 nm. The calibration curve was established with gallic acid at 10 and 200 mg/L concentrations. Total phenolics were expressed as mg gallic acid equivalent per 100 g of freeze-dried petal weight (mg GAE/100 g DW) [22]. Finally, using the solvent and time that maximized the extraction of phenolics, the rest of the samples under investigation were extracted according to the above process.

To identify individual phenolics, 20 µL of the methanolic extract was applied to an Agilent 1200 series RRLC with a DAD-UV-Vis detector operating between 220 and 500 nm and a Zorbax Eclipse Plus C18 column (4.6 × 150 mm, 5 µm) (Agilent Technologies, Santa Clara, CA, USA) at 30 °C [5]. The mobile phase was a linear gradient at a flow rate of 1 mL/min of an aqueous solution of formic acid (0.01%) (solvent A) and acetonitrile (solvent B). During the run, the mobile phase was pumped as follows: 100% A at 0 min; 95% A + 5% B at 5 min; 50% A + 50% B at 20 min; washing and re-equilibration of the column at 30 min. Spectra stored in the Open Lab ChemStation software (version 2.15.26) were used to identify the individual phenolics. In contrast, calibration curves were used for quantification at concentrations ranging from 0. 33 to 1 mg/mL of shikimic acid, benzoic acids (*p*-hydroxybenzoic acid, 3-hydroxybenzoic acid, 2-methoxybenzoic acid, 3-methoxybenzoic acid, 2,5-dihydroxybenzoic acid, gallic acid, vanillic acid, syringic acid, *p*-coumaric acid, *o*-coumaric acid, *m*-coumaric acid), hydroxycinnamic acids (caffeic acid, chlorogenic acid, ferulic acid), flavonols (kaempferol, quercetin, quercetin glycoside, rutin), and flavones (chrysin, luteolin, flavanones (naringin)). Each phenolic compound was expressed as milligrammes per hundred grammes of freeze-dry petal weight (mg/100 g DW). Total phenolics were calculated as the sum of the individual compounds.

### 2.5. Quantification of Total Anthocyanins

To extract anthocyanins from the freeze-dried flower powder, 20 mg of freeze-dried powder was weighed and mixed with 2 mL of absolute ethanol. The mixture was shaken for 3 min in an ultrasonic bath. Residual solids were removed by centrifugation at 1400 rpm for 5 min at 4 °C [23,24,25]. Quantification of anthocyanins was performed using the pH differential method, which is widely used for this type of analysis. For the procedure, 50 μL of the extract was diluted with 200 μL of a 0.025 M potassium chloride buffer at pH 1, while 50 μL of the extract was mixed with 200 μL of a 0.4 M sodium acetate buffer at pH 4.5 [26,27]. The absorbances of each dilution were measured at 520 nm and 700 nm in a spectrophotometer with a microplate reader. The calibration curve was constructed over a 0.05 to 0.2 mg/mL concentration range. The total anthocyanin concentration was expressed as mg cyanidin-3-glucoside chloride per gram dry weight (mg C-3-gl/g DW).

### 2.6. Quantification of Organic Acids

Organic acids were extracted from 40 mg of lyophilised powder with 1.5 mL of 0.02 N sulphuric acid containing metaphosphoric acid (0.05%) and *DL*-homocysteine (0.02%). The mixture was homogenized and vortexed under ultrasound for 3 min, and 500 μL of deionised water was added. The supernatant was collected by centrifugation at 1400 rpm for 5 min at 4 °C and filtered through a 0.45 µm PVDF filter. Separation of organic acids was performed using 10 µL of the extract applied to an Agilent 1200 series RRLC, DAD-UV-Vis detector at 210 nm, and a YMC-Triart C18 column (150 × 4.6 mm, 3, 12 nm, 400 bar) (YMC Europe GmbH, Dinslaken, Germany) at 30 °C. The mobile phase was a 0.027% sulphuric acid solution pumped at 1 mL/min for 30 min. Organic acids were analyzed using spectra stored in Open Lab ChemStation software and retention time. Calibration curves with concentrations between 33.3 and 100 mg/mL of citric acid, malic acid, and L-(+)-tartaric acid standards were used for quantification [18]. Each organic acid was expressed in grammes per 100 g of freeze-dried petal weight (g/100 g DW).

### 2.7. Antioxidant Activity (ABTS and DPPH)

To extract the ABTS antioxidant activity assay sample, 20 mg of lyophilised powder was mixed with 400 µL of methanol and 400 µL of distilled water separately. The mixture was homogenized by vortexing and shaken in an ultrasonic bath for 3 min. Afterwards, the solution was separated by microcentrifugation at 14000 rpm for 5 min at 4 °C. The resulting solid was mixed separately with 560 µL of acetone and 240 µL of distilled water. The process was repeated to obtain the supernatant, which was then combined with the previous supernatant. The final mixture was refrigerated until quantification.

To prepare the ABTS^•+^ radical, a 1:1 solution of 7 mM ABTS was mixed with 2.45 mM potassium persulfate and left to stand in the dark for 16 h. The resulting ABTS^•+^ radical solution was diluted approximately 1/10 with absolute ethanol or until an absorbance of 0.7 at 754 nm was achieved [20]. For the calibration curve, a stock solution of 0.25 mM Trolox was prepared and diluted to 0.01 to 0.07 mM concentrations. To quantify the samples, 20 µL of the final supernatant solution was added to a 96-well VWR tissue culture plate (Novachen, Connersville, IN, USA). Subsequently, 280 µL of the ABTS^•+^ radical solution was added to each well following the procedure described by Chan et al. [28]. The absorbance was measured at 754 nm using a spectrophotometer, specifically a Thermo Scientific Multiskan GO microplate reader (Agilent Scientific Instruments, Santa Clara, CA, USA). Finally, the antioxidant activity was expressed as mmol Trolox equivalents per gram of freeze-dried petal weight (mmol TE/g DW).

To extract the DPPH antioxidant activity assay sample, 20 mg de lyophilised powder was mixed with 2 mL of methanol. The mixture was homogenized by vortexing and shaken in an ultrasonic bath for 3 min. Afterwards, the solution was separated by microcentrifugation at 14,000 rpm for 5 min at 4 °C. For the calibration curve, a stock solution of 0.25 mM Trolox was prepared and diluted to 0.01 to 0.07 mM concentrations. To quantify the samples, 20 µL of the final supernatant solution was added to a 96-well VWR tissue culture plate. Subsequently, 280 µL of the DPPH^•^ (10 mg of DPPH in 50 mL of methanol) was added. The absorbance was measured at 560 nm after 30 min [29]. Antioxidant activity was expressed as mmol Trolox equivalents per gram of freeze-dried petal weight (mmol TE/g DW).

### 2.8. Antimicrobial Activity

The extract was prepared by weighing 2 g of the freeze-dried sample of the 35 species with the largest sample size and mixing it with 10 mL of 50% ethanol. The mixture was homogenized and vortexed in an FS60 ultrasonic bath (Fisher Scientific, USA) for 6 min. The resulting supernatant was collected by centrifugation at 14,000 rpm for 3 min using a microcentrifuge (Eppendorf, Germany). This extraction procedure was performed twice, with 10 mL of the ethanol solution for each extraction. The final supernatant was filtered through PDVF filters with a pore size of 0.45 µm and a diameter of 25 mm. The extract was dried using a Christ Alpha 1-4 LDplus freeze dryer (GmbH, Germany). Finally, the dried extract was reconstituted in 1 mL of sterile distilled water to evaluate antimicrobial activity using the well diffusion method according to Clinical and Laboratory Standards Institute (CLSI) guidelines with some modifications [30,31,32].

The antibacterial properties of the flower extracts were evaluated against Gram-positive bacteria such as *Staphylococcus aureus* ATCC 6538P and Gram-negative bacteria such as *Escherichia coli* ATCC 8739, *Pseudomonas aeruginosa* ATCC 9027, and *Streptococcus mutans* ATCC 25175. In addition, antifungal activity was evaluated against two pathogenic fungal species, *Candida albicans* ATCC 1031 and *Candida tropicalis* CC 13803. All bacterial strains used in this study were obtained from the American Type Culture Collection (ATCC, Manassas, VA, USA) and maintained at −80 °C in a 25% (*v*/*v*) glycerol solution. Both Gram-positive and Gram-negative bacteria were pre-cultured in Brain Heart Infusion (BHI) overnight at 37 °C on a rotary shaker. Each strain was then calibrated to a concentration of 0.5 MacFarland standard (10^8^ cells/mL). The fungal inoculum was obtained from a 24 h culture of the isolated fungal strains in Yeast Peptone Dextrose Broth (YPDB). Each strain was then adjusted to the 0.5 MacFarland standard (resulting in a final concentration of 10^6^ cells/mL).

The agar well diffusion method was used to evaluate the antimicrobial activity of the flower extracts. The suspension of active microorganisms was spread evenly on solidified Müller-Hinton agar (MHA) for bacterial strains and on Sabouraud dextrose agar (SDA) for fungal strains using a sterile swab. 5 mm diameter agar wells were prepared using a sterile punch in each plate. Then, 80 µL of flower extracts were added to the wells, and the Petri dishes were incubated at 37 °C/18 h for bacteria and 35 °C/48 h for fungi. The zone of inhibition obtained was measured in millimetres. Streptomycin and fluconazole were used as controls for growth inhibition at recommended working concentrations for the bacterial and fungal strains, respectively. In addition, distilled water was used as a negative control. These assays were performed in, at least, triplicate.

### 2.9. Statistical Analysis

Statistical analysis was conducted using Statgraphics Centurion XVII, Rstudio, and Sigmaplot 14.0 software. Results are given as the mean ± standard deviation. A simple ANOVA was employed to identify significant differences, with a significance level set at *p* < 0.05. Furthermore, correlation and principal component analyses explored potential relationships among the study parameters—this analysis aimed to uncover any associations between the variables under investigation.

## 3. Results

### 3.1. Physicochemical Quantification

Table 2 shows the results of the physicochemical analyses on the petals studied. This study evaluated weight, size, pH, soluble solids, titratable acidity, moisture, and ash.

Flower weights ranged from 0.01 g in *P. vulgaris* and *L. rugulosa* to 72.79 g in *X. ro-bustum*. Concerning flower size, longitudinal and equatorial diameters displayed notable variations, with values ranging from 1.07 cm in *L. rugulosa* to 25.30 cm in *I. hancockii* and from 0.36 cm in *O. cuspidatum* and *P. vulgaris* to 62.85 cm in *A. purpurata*. The pH levels ranged from 2.00 in *A. poiretii, B. macrophylla*, and *I. walleriana* to 11.40 in *P. urvilleana*. Soluble solids ranged from 0.21 °Brix in *P. fragrantissimum* to 15.80 °Brix in *P. lutea*. The total titratable acidity spanned from 0.05% in *X. robustum* and *H. episcopalis* to 3.81% in *A. corymbosa*. Moisture content varied from 14.26% in *M. sodiroana* to 96.05% in *I. walleriana*. The ash content ranged from 0.03% in *I. hancockii* to 8.02% in *S. oblonga*. 

### 3.2. Optimization of Extraction Parameters and Quantification of Carotenoids

**Figure 2:**
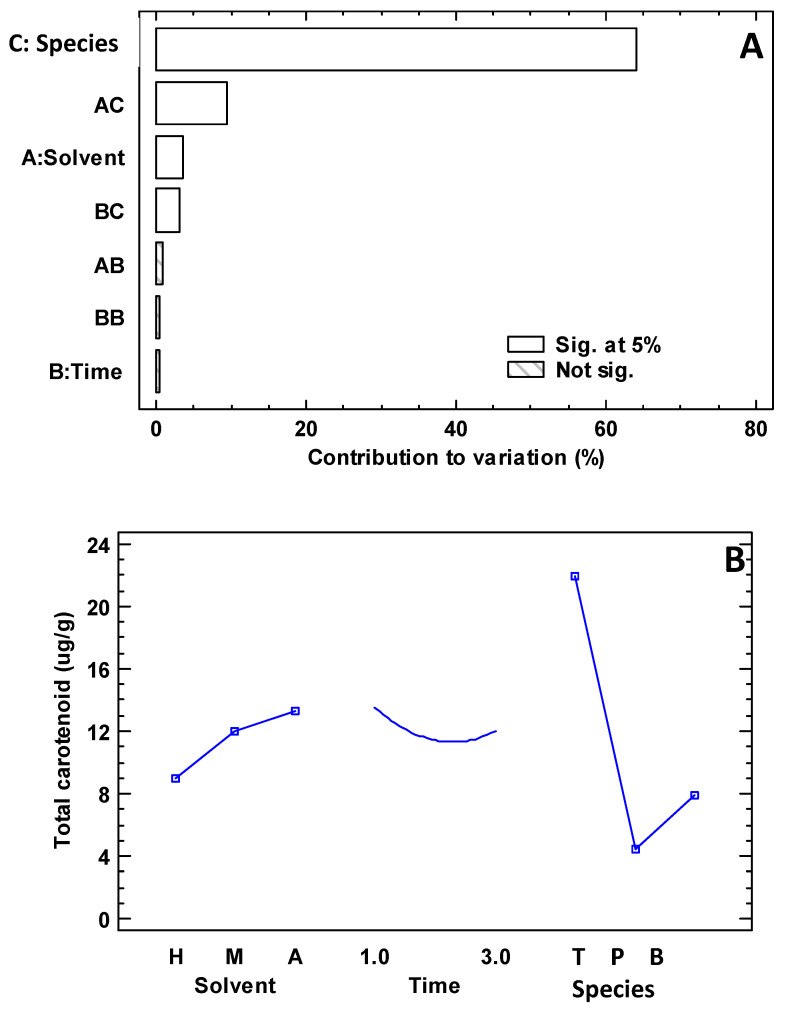
Results of the experimental design for the extraction of carotenoids. Note: In (**A**), the capital letters on the y-axis represent: A, solvent; B, time; C, species. In (**B**), the capital letters on the x-axis represent: H, *n*-hexane–acetone (1:1); M, methanol–acetone–dichloromethane (1:1:2); A, acetone–methanol (2:1); time in minutes; T, *Taraxacum officinale*; P, *Pyrostegia venusta*; B, *Buddleja globose*.

Figure 2A,B shows the experimental design results for the carotenoids’ micro-extraction. The solvents selected for optimization in this study were derived from a literature review of 119 species and previous studies [9,33].It is highlighted that both the plant species and the solvent used for extraction have a significant influence, with extraction time being a minor effect compared to the other variables. It is important to note that the maximum carotenoid extraction was obtained using the solvent combination of methanol–acetone–dichloromethane (1:1:2) and acetone–methanol (2:1), with an extraction time of one minute.

Table 3 shows the analysis results to quantify the total carotenoid content in various flower species. The results show a significant variation in the levels of total carotenoids among the multiple species examined. The recorded values ranged from 0.25 mg β-carotene/g DW in *C. cuatrecasasii*, which exhibited a pink visual colour, to 292.5 mg β-carotene/g DW in *R. alpinia*, characterized by a visible orange colour. These results highlight the diverse and substantial differences in carotenoid accumulation within the studied flower species, emphasizing the influence of pigmentation on carotenoid content.

### 3.3. Optimization of Extraction Parameters and Quantification of Phenolic Compounds

**Figure 3:**
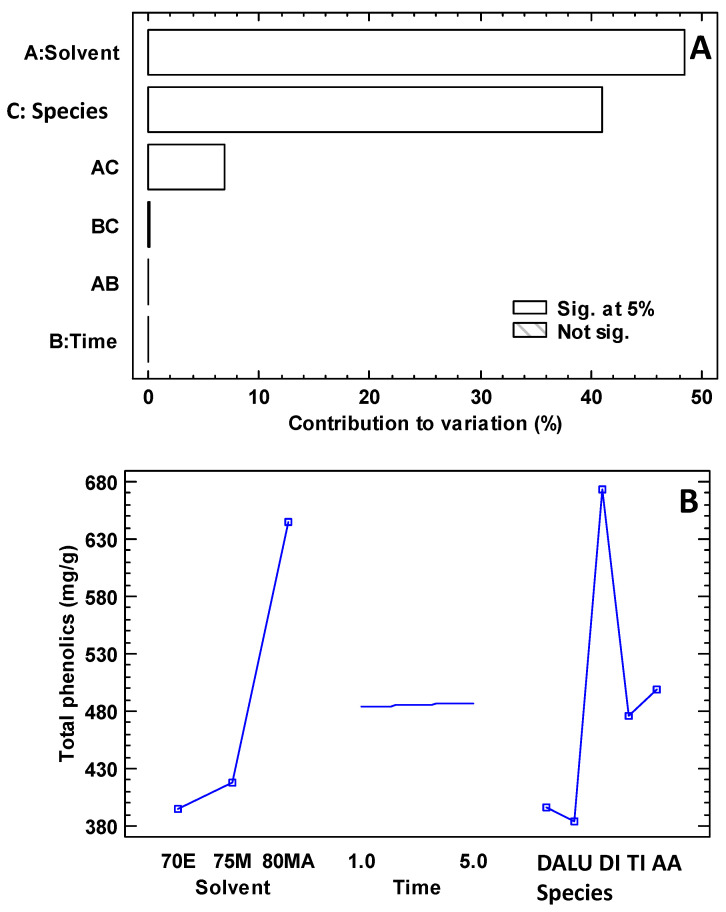
Results of the experimental design for the extraction of phenolics. Note: In (**A**), the capital letters on the y-axis represent: A, solvent; B, time; C, species. In (**B**), the capital letters on the x-axis represent: 70E, 75% ethanol; 75M, 75% methanol; 80MA, 80% methanol acidified with 0.1% hydrochloric acid; time in minutes; DA, *Dahlia pinnata*; LU, *V*; DI, *Dianthus caryophyllus*; TI, *Plerona urvilleanum*; AA, *Agapanthus africanus*.

Figure 3A,B show the experimental design results applied to the phenolic compound micro-extraction. The solvents selected for optimization in this study were derived from a literature review of 119 species and previous studies [9,33]. It is highlighted that both the solvent and the plant species used for extraction have a significant influence, with extraction time being a minor effect compared to the other variables. Notably, the maximum phenolics extraction was obtained using 80% methanol acidified with 0.1% hydrochloric acid, with an extraction time of five minutes. The concentration of total phenolics determined by the spectrophotometric method ranged from 50.60 mg GAE/g DW in *S. stromanthoides*, which had an orange visual colour, to 510.53 mg GAE/g DW in *P. urvilleana*, characterized by a yellow-orange visual colour (Table 3).

Table 4 shows the concentration of gallic acid (ranging from 3.6 mg/100 g DW in *G. ichtyoderma* to 869.4 mg/100 g DW in *C. cornutus*), protocatechuic acid (ranging from 4.6 mg/100 g DW in *H. coronarium* to 231.0 mg/100 g DW in *P. angustifolia*.), *p*-coumaric acid (ranging from 30.2 mg/100 g DW in *H. wagneriana* to 2929.0 mg/100 g DW in *C. bracteata*), *m*-coumaric acid (ranged from 30.2 mg/100 g DW in *H. wagneriana* to 2929.0 mg/100 g DW in *C. bracteata*), *m*-coumaric acid (ranged from 23.5 mg/100 g DW in *I. hancockii* to 12,044.0 mg/100 g DW in *A. poiretii*), syringic acid (ranged from 11.0 mg/100 g DW in *S. stromanthoides* to 3225.0 mg/100 g DW in *A. poiretii*), chlorogenic acid (ranged from 37.1 mg/100 g DW in *M. erythrophylla* to 3435.0 mg/100 g DW in *S. dulcamara*), 4-hydroxybenzoic acid (range from 17.6 mg/100 g DW in *S. oblonga* to 10,729 mg/100 g DW in *E. arborea*), caffeic acid (range from 108. 6 mg/100 g DW in *I. hancockii* to 5893.0 mg/100 g DW in *A. corymbosa*), ferulic acid (ranged from 148.2 mg/100 g DW in *I. hancockii* to 5849.0 mg/100 g DW in *T. grandiflora*), rutin (ranged from 16. 9 mg/100 g DW in *H. episcopalis* to 3814.0 mg/100 g DW in *C. angustifolia*), quercetin (ranged from 28.1 mg/100 g DW in *H. latispatha* to 5119.0 mg/100 g DW in *D. pinnata*), quercetin glucoside (ranged from 32.8 mg/100 g DW in *I. triloba* to 2950.0 mg/100 g DW in *L. rugulosa*), and kamferol (ranged from 39.4 mg/100 g DW in *H. coccineum* to 8236.0 mg/100 g DW in *D. pinnata*). The total concentration as the sum of the individual phenolics ranged from 6.3 mg/100 g DW in *R. alpinia* to 110364 mg/100 g DW in *M. erythrophylla*. These results show the diversity in the accumulation of phenolic compounds within the flower species studied.

### 3.4. Quantification of Total Anthocyanins

Anthocyanins are a subclass of flavonoids [9]. Table shows the results of the quantified analysis of the content of total anthocyanins in different species of flowers. Thus, the concentrations ranged from 0.03 mg C-3-gl/g DW (*C. nobilis*) to 7.35 mg C-3-gl/g DW (*P. heteromallum*). In addition, species such as *B. macrophylla* (2.44 mg C-3-gl/g DW), *D. balsapampae* (2.04 mg C-3-gl/g DW), *H. rosa-sinensis* (1.93 mg C-3-gl/g DW), and *D. pinnata* (1.84 mg C-3-gl/g DW) also showed high anthocyanin concentrations.

### 3.5. Quantification of Organic Acids

Table 3 shows the results of the quantified analysis of the content of organic acids in different species of flowers. The data show a significant variation in the content of these compounds among the species studied. Citric acid content ranged from 20.2 mg/100 g DW in *T. grandiflora* to 17,819 mg/100 g DW in *H. rosa-sinensis*, the latter being particularly high. For malic acid, values ranged from 13.2 mg/100 g DW in *B. arborea* to 16,614 mg/100 g DW in *A. piretii.* Finally, tartaric acid showed values ranging from 4.8 mg/100 g DW in *C. spiralis* to 5488 mg/100 g DW in *H. latispatha*. These results show the remarkable diversity in the composition of organic acids among the flower species analyzed, highlighting their potential for specific applications based on their biochemical profile.

### 3.6. Antioxidant Activity (ABTS and DPPH)

Table 3 presents the results of the percentage inhibition of the ABTS radical and the corresponding antioxidant activity using aqueous methanol and acetone extracts of different flower species. The antioxidant activity by the ABTS method, expressed in concentration, ranged from 2.02 mmol ET/g DW in *G. nicaraguensis* to 7.80 mmol TE/g DW in *T. floribunda*. In addition, *H. rosa-sinensis* (7.67 mmol TE/g DW), *A. lepidotus* (7.43 mmol TE/g DW), and *I. hancockii* (7.33 mmol TE/g DW) also showed high antioxidant activity values. In turn, the antioxidant activity of the DPPH method ranged from 14.98 mmol TE/g DW (*H. latispatha*) to 77.77 mmol TE/g DW (*I. hawkeri*). In addition, species such as *B. americana* (75.87 mmol TE/g DW), *I. walleriana* (73.23 mmol TE/g DW), *A. corymbosa* (67.63 mmol TE/g DW), and *D. pinnata* (65.79 mmol TE/g DW) showed a high antioxidant concentration.

### 3.7. Antimicrobial Activity

**Figure 4:**
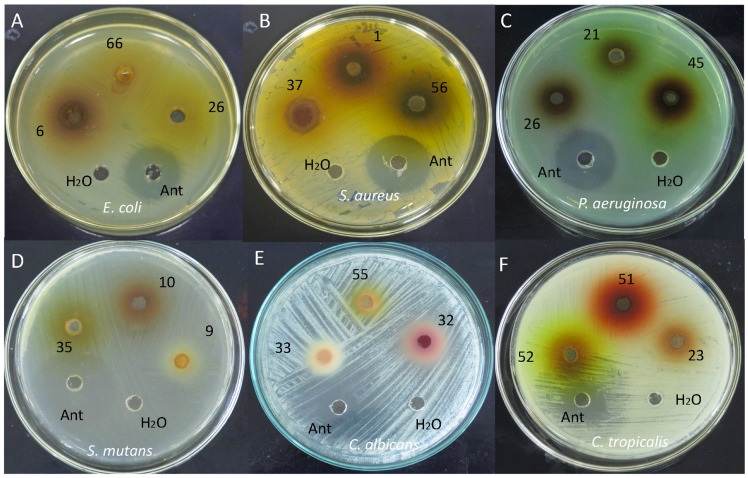
Antimicrobial activity of flower extracts against (**A**) Escherichia coli; (**B**) Staphylococcus aureus; (**C**) Pseudomonas aeruginosa; (**D**) Streptococcus mutans; (**E**) Candida albicans; (**F**) Candida tropicalis. Note: 1, *M. erythrochlamys*; 6, *C. argentea*; 9, *P. fragrantissimum*; 10, *P. strictum*; 21, *G. nicaraguensis*; 22, *R. veitchii*; 23, *C. cornutus*, 26, *C. spiralis*, 27, *C. spiralis*; 32, *A. poiretii*; 33, *B. macrophylla*; 35, *E. americana*; 37, *G. ichthyoderma*; 45, *S. stromanthoides*; 51, *B. spectabilis*; 52, *E. aurantiacus*; 55, *M. erythrophylla*; 56, *M. philippica*, 66, *H. coccineum*.

Antimicrobial susceptibility testing is critical for the effective management of pathogenic microorganisms. The well diffusion method demonstrated the magnitude of susceptibility of the pathogenic microorganisms (Figure 4). Thus, Table 5 presents the inhibition zone measurements for the antimicrobial activity of various flower extracts against bacterial and fungal strains. The inhibition zones ranged from 0 mm, indicating no activity in several species, to a maximum of 22.0 mm in *B. spectabilis*, which exhibited significant antibacterial efficacy. This variability highlights the broad spectrum of antimicrobial activity across the flower species tested. Notably, the extracts exhibited no antifungal activity against the tested fungi, except *S. heteromallum*, which displayed selective inhibition against *Candida albicans*. These findings suggest that while several floral species demonstrate potential as antibacterial agents, their antifungal properties are more limited and species-specific.

### 3.8. Statistical Analysis

**Figure 5:**
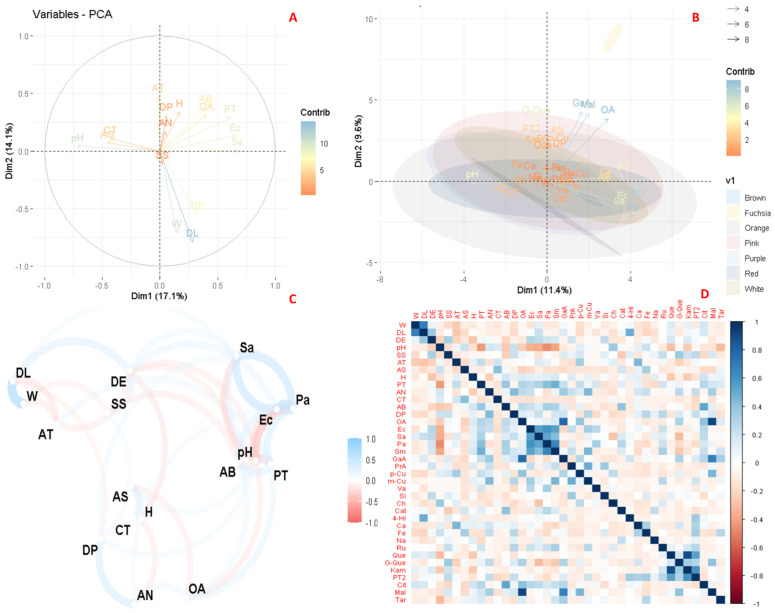
Exploratory multivariate analysis using principal components (**A**,**B**), and correlation analysis (**C**,**D**) of physicochemical parameters, bioactive compounds, and antioxidant activity of the floral species analyzed. Note: W, weight; DL, longitudinal diameter; DE, equatorial diameter; SS, soluble solids; AT, titratable acidity; H, humidity; AS; ash; CT, total carotenoid; PT, total phenolics; AB, antioxidant activity of ABTS, DP, antioxidant activity of DPPH, OA, organic acids; Ga, Gallic acid; Pr, protocatechuic acid; p-Cu, *p*-coumaric acid; o-Cu, *o*-coumaric acid; Va; vanillic acid; Si, syringic acid; Ch, chlorogenic acid; Cat, caffeic acid; 4-Hi, 4-hydroxy-benzoic acid; Ca, caffeic acid; Fe, ferulic acid; Na, naringin; Ru; rutin; Que, quercetin; G-Que, quercetin glucoside; Kam, kaempferol; PT2, total phenolics; Cit, citric acid; Mal, malic acid; Tar, tartaric acid; Ec, *Escherichia coli*; Sa, *Staphylococcus aureus*; Pa, *Pseudomonas aeruginosa*; Sm, *Streptococcus mutans*; Ca, *Candida albicans*; Ct, *Candida tropicalis*.

Figure 5A shows the principal component analysis (PCA) considering the visual colour of the flowers analyzed, while Figure 5B shows the PCA considering the total set of variables in the study. Figure 5C shows a correlation plot including all variables considered in the study, and Figure 5D shows the specific correlation plot for these variables.

## 4. Discussion

### 4.1. Physicochemical Quantification

The flower in this study varied widely in shape, size, and weight, which are characteristics of each species. For example, *X. robustum*, which had the highest weight (72.79 g), is a species with very fleshy and spongy flowers [34], which gives it a high weight. In addition, these morphological characteristics are related to the degree of maturity, which is why in this study we considered fully developed (open) flowers, as they have the highest weight and size, as suggested by others [35]. In turn, the characteristics are also influenced by plant size, age, tissue water content, inherent species variability, and environmental influences [36,37]. For instance, the literature data suggest that *H. rosa-sinensis*, typically found in tropical and subtropical regions, exhibits a red variety with lengths of up to 15 cm [38,39,40]; however, the present study recorded a value of 24.43 cm. Another study focusing on the genus *Heliconia* reveals that tropical species such as *H. latispatha*, *H. wagneriana*, and *H. rostrata* have bract sizes ranging from 12 to 14 cm, 11 to 13 cm, and 6.5 to 7.5 cm, respectively [41]. This study’s corresponding values were 11.20 cm, 12.38 cm, and 8.51 cm, respectively. Conversely, *O. cuspidatum*, commonly found in sub-deciduous and deciduous tropical forests, typically exhibits a red cluster-shaped inflorescence measuring 2.1 to 3.5 cm in length, surpassing the values found in this study (1.69 cm) [42]. A wide range of pH values was observed, from very acidic to slightly alkaline samples, reflecting the influence of various chemical and biological processes within these species, including nutrient absorption, metal availability, and enzymatic activity [35]. The variation in pH can be attributed to the specific characteristics of the soil in which the plants are grown and the adaptive strategies employed by each species. For instance, species like *C. nobilis* and *T. floribunda* from the Ericaceae family thrive in slightly acidic soils abundant in organic matter, typically found near the Antisana volcano in Ecuador [43]. On the other hand, *M. erythrochlamys*, belonging to the Acanthaceae family, grows in clay soil with a pH close to neutral [44], resulting in flowers with a pH of 4.00 and 9.15.

The species *P. lutea* (15.8 °Brix), *B. macrophylla* (12.00 °Brix), and *Oncidium* sp.(|11.20 °Brix) showed a notably high concentration of soluble solids, even exceeding the soluble solids content of fruits such as watermelon (*Citrullus lanatus)*, where values of 10.43 to 13.56 °Brix have been reported [45]. In this sense, soluble solids include the ratio between sugars, organic acids, and other soluble compounds, which play an essential role in plant metabolism as a source of energy and are also responsible for the taste and quality of plant species. Variations in their content may reflect plants’ different chemical composition and degree of maturity [46,47].

In terms of total titratable acidity, *A. corymbose* (3.81%), *C. mollis* (2.8%), and *P. heteromallum* (2.73%) showed the highest concentration. Total titratable acidity is a quantitative measure of the organic acids present in a plant species, such as citric acid, malic acid, tartaric acid, and other acidic compounds. These compounds can significantly affect plant species’ flavour, shelf life, and stability. Variations in titratable acidity values can be attributed to plants’ unique metabolic and physiological characteristics, environmental factors, and the stage of flower development [46,47]. However, it is essential to note that this study specifically focused on fully developed flowers, as titratable acid content tends to be higher, as demonstrated in a study examining different stages of feijoa (*Acca sellowiana*) flower development [47].

Species such as *I. walleriana* (96.05%), *I. hawkeri* (95.70%), and *H. coronarium* (94.70%) showed a high moisture content. This parameter is critical to plant growth and development by influencing water availability and transpiration. Optimal moisture levels are crucial for the efficient functioning of physiological processes in plants [48]. In turn, literature sources showed that *H. rosa-sinensis* had an average moisture content of 90%, which is consistent with the results of this study, where a moisture content of 91% was reported. In addition, species such as *S. oblonga* (8.02%), *H. coccineum* (6.12%), and *A. purpurata* (4.02%) showed high ash concentrations. This parameter indicates the percentage of inorganic residues remaining after combustion and provides information on the mineral composition of the plants, which may vary between cultivars [48].

### 4.2. Optimization of Extraction Parameters and Quantification of Carotenoids

Carotenoids are plant pigments responsible for photosynthesis. Carotenoids are essential for plant health because they act as antioxidants and increase resistance to adverse conditions such as ultraviolet radiation and oxidative stress. These secondary metabolites protect against oxidative stress and benefit human health. e.g., β-carotene is a precursor to vitamin A and has antioxidant properties [9]. Thus, the efficacy of carotenoid extraction was significantly influenced by the type of solvent used and the plant species. The highest extraction was obtained using the mixtures of methanol–acetone–dichloromethane (1:1:2) and acetone–methanol 2:1, with an extraction time of 1 min, especially for the species *T. officinale.* These results align with previous research by other scientists, who have also highlighted the importance of the choice of solvent in the extraction of carotenoids to ensure accurate quantification of these compounds [33].

Species such as *R. alpinia* (orange visual colour), *A. papposa* (yellow visual colour), *D. balsapampae* (yellow visual colour), and *H. coccineum* (orange visual colour) showed remarkably elevated levels of carotenoids, with values of 1925.0 mg β-carotene/g DW, 211.00 mg β-carotene/g DW, 129.43 mg β-carotene/g DW, and 126.26 mg β-carotene/g DW, respectively. These variations can be attributed to species genetics, growth conditions, exposure to sunlight, and environmental influences [5].

Species such as *X. robustum* (white visual colour), *H. latispatha* (red visual colour), and *C. cuatrecasasii* (pink visual colour) showed low concentrations of total carotenoids, with values of 0.59 mg β-carotene/g DW, 0.43 mg β-carotene/g DW, and 0.25 mg β-carotene/g DW, respectively. It is worth noting that the literature reports a total carotenoid content of 162.00 µg/g fresh weight for *H. rosa-sinensis* cultivated at the Cairo Faculty of Agriculture [49]. In contrast, the present study reports a higher concentration (3.64 mg β-carotene/g DW). The difference in concentration may be due to differences in cultivation conditions, as the study in question used average environmental conditions of 35 °C and 40% relative humidity. In contrast, this study collected samples from Ecuador’s species’ natural habitat, with an average temperature of 25 °C and relative humidity between 85 and 90%. Furthermore, an investigation into *A. cathartica* demonstrated varying carotenoid concentrations across different plant parts, with 9.01 mg/g in flowers, 12.41 mg/g in leaves, 4.53 mg/g in roots, and 2.91 mg/g in shoots [50]. This shows the significant influence of the analyzed plant part on the total carotenoid content. In this study, the concentration in the petals of *A. cathartica* was found to be 32.53 mg β-carotene/g DW.

In turn, *R. alpinia* presented the highest concentrations of total carotenoids compared to the other floral species studied [5]. These results are of great importance for various industries, as they may lead to the creation of new products, as evidenced by the literature, which mentions the use of this species in the ancestral treatment of fevers caused by snake bites and to relieve the pain of bruises [33]. It is also important to note that the Zingiberaceae family, to which *R. alpinia* belongs, is characterized by leaves with antifungal, cytotoxic, anti-inflammatory, antipyretic, antioxidant, insecticidal, hepatoprotective, and immunomodulatory properties [51].

### 4.3. Optimization of Extraction Parameters and Quantification of Total Phenolic Compounds

The efficiency of the phenolic extraction was significantly influenced by the type of solvent and the plant species used. It is evident that the highest extraction was obtained using the 80% methanol solution acidified with 0.1% hydrochloric acid, with an extraction time of 3 min, particularly in the case of the species *D. caryophyllus*. These results align with previous studies by other researchers, who have also highlighted the importance of the choice of solvent in the phenolic extraction process to ensure accurate quantification of these compounds [19]. Thus, a previous study reported total phenolic concentrations in *H. rosa-sinensis* grown in Cairo using different solvents. The concentrations ranged from 186.17 mg GAE/100 g FW, 235.77 mg GAE/100 g FW, and 281.23 mg GAE/100 g FW using absolute ethanol, water, and 80% ethanol, respectively [49]. In contrast, this study determined a concentration of 290.3 mg GAE/g DW using an acidified methanolic solution.

Some species are characterized by high levels of total phenolics; e.g., *T. sorensis*, *P. urvilleana*, and *C. mollis* exhibited remarkable concentrations of 581.48 mg GAE/g, 510.53 mg GAE/g, and 354.2 mg GAE/g, respectively. These species can be considered rich sources of phenolic compounds, as phenolics are widely known for their remarkable antioxidant properties and potential benefits for human health. These compounds function as potent defenders against oxidative stress and have been suggested to play a role in preventing chronic diseases like heart, cancer, and neurodegenerative disorders [33]. At the same time, other species showed lower levels of total phenolics; e.g., *S. stromanthoides*, *H. episcopalis*, and *R. veithchii* recorded levels of 50.6 mg GAE/g, 56.86 mg GAE/g, and 72.62 mg GAE/g, respectively. In this regard, the variation in total phenolic content observed among the different species may be due to genetic and environmental factors, soil type, nutrient availability, exposure to sunlight, and other environmental conditions, as suggested by other authors [5]. In this regard, a study on *H. rostrata* flowers identified the presence of phenolics through phytochemical screening, with a concentration of 155.21 mg GAE/100 g DW [9]. Similarly, in the case of *C. argentea*, a study reported a concentration of 47.00 mg GAE/100 mg using an ethanolic solution of flower extract [52], whereas in this study, a concentration of 140.63 mg GAE/g DW. Another investigation on *A. cathartica* reported a concentration of 2839 µM/g in flowers, 11,906 µM/g in leaves, 19344 µM/g in roots, and 3455 µM/g in shoots [50], whereas in this study a concentration of 167.12 mg GAE/g DW was observed.

Certain species in this study showed significant concentrations of individual phenolic compounds, e.g., in gallic acid, the species *C. cornutus* (869.4 mg/100 g DW), in protocatechuic acid the species *P. angustifolia* (231.4 mg/100 g DW), in *p*-coumaric acid the species *C. bracteate* (2929 mg/100 g DW), in *m*-coumaric acid and syringic acid the species *A. poiretii* with a concentration of 12,044 mg/100 g DW and 3225 mg/100 g, respectively, in chlorogenic acid the species *S. dulcamara* (3435 mg/100 g DW), in 4-hydroxybenzoic acid the species *B. arborea* (10,729 mg/100 g DW), in caffeic acid the species *A. corymbose* (5893 mg/100 g DW), in ferulic acid the species *T. grandiflora* (5848 mg/100 g DW), for quercetin glucoside the species *L. rugulosa* (2950 mg/100 g DW), and for kaempferol the species *D. pinnata* (8236 mg/100 g DW). In this context, information was presented on the widespread use of *C. cornutus* for lymphatic disorders, canker sores, and cold sores [6]. It was reported that various parts of the non-traditional shrub of *C. bracteate* had been traditionally used in nutrition and the roots to control diarrhea and diabetes [53]. *A. poiretii* has been shown to have a beneficial effect on the homeostatic mechanisms [54]. *p*-Coumaric, caffeic acid and ferulic acids are phenolic compounds with antimicrobial properties against various microorganisms when used in polymeric food packaging materials [55].

### 4.4. Quantification of Total Anthocyanins

Anthocyanins are a type of phenolic compound in the flavonoid group. They are responsible for the colours of many flowers, which contribute to their beauty, and they offer health benefits thanks to their antioxidant properties. In this regard, studies on flowers have shown that anthocyanins are predominant in dahlias, with variations in concentration between varieties (1.14 mg cyanidin-3-*O*-glucoside chloride/g in the variety ‘Colorado Classic’ and 1.25 mg cyanidin-3-*O*-glucoside chloride/g in the variety ‘La Baron’) [56]. These values were lower than those reported in this study for *D. pinnata* (1.84 mg C-3-gl/g dry weight). Furthermore, in *Hibiscus rosa-sinensis*, the concentration in the extract was 0.46 mg cyanidin-3-glucoside/g [57], which was lower than the value reported in this study (1.93 mg C-3-gl/g dry weight).

### 4.5. Quantification of Organic Acids

The results of the analysis of organic acids in flower species reveal considerable variation between species, highlighting the inherent biochemical diversity of each species and their contribution to the nutritional value and functional properties of flowers. These properties are increasingly recognized for their health benefits. A prominent example is *H. sabdariffa*, which contains citric, malic, and tartaric acids, all contributing to its diverse pharmacological effects [58]. The variability in the contents of these acids may be influenced by factors such as genetics, growing environment, flower maturity, and environmental conditions such as light and soil type [59,60].

In this study, several species showed high concentrations of organic acids. Citric acid, which plays a vital role in the tricarboxylic acid cycle in plants [61], showed a wide range of concentrations among the species tested. The highest concentrations were observed in *H. rosa-sinensis* (17,818 mg/100 g DW), *H. wagneriana* (3800 mg/100 g DW), *T. floribunda* (3516 mg/100 g DW), *E. americana* (2874 mg/100 g DW), and *I. gesnerioides* (2662 mg/100 g DW). These results suggest that these species can accumulate high levels of organic acids, favouring their possible use in food and medicinal applications as natural acidifying agents.

For malic acid, known for its role in plant metabolism [61], significant variability was also observed. Species such as *A. piretii* (15,613 mg/100 g DW), *C. cornutus* (11,655 mg/100 g DW), *A. lepidotus* (5591 mg/100 g DW), *E. aquaticum* (4605 mg/100 g DW), *P. urvilleanum* (3540 mg/100 g DW), and *P. angustifolia* (3121 mg/100 g DW) stood out for their high content of this acid, positioning them as potential sources of malic acid in industrial and food applications.

Tartaric acid, generally found in lower concentrations in most plant species [61], also showed considerable variability among the flowers analyzed. The species with the highest concentrations of tartaric acid were *H. latispatha* (5488 mg/100 g DW), *B. spectabilis* (3197 mg/100 g DW), *B. americana* (1519 mg/100 g DW), *R. veitchii* (836 mg/100 g DW), and *C. mollis* (708 mg/100 g DW). Tartaric acid is known to have antioxidant properties, suggesting that these species may offer additional health benefits to consumers by providing a natural source of antioxidants [62].

### 4.6. Antioxidant Activity (ABTS and DPPH)

In this study, aqueous solutions of methanol and acetone were used to extract both water-soluble and liposoluble compounds associated with antioxidant activity. Since organic compounds have different polarities and solubilities, no single extraction method is optimal for all types of molecules, such as phenolic compounds and carotenoids [63,64,65]. This variability affects the extraction efficiency and the interaction of the antioxidant compounds with the ABTS and DPPH radical in the antioxidant activity assay. For example, solvents such as ethanol and methanol effectively extract phenolic compounds and flavonoids, whereas acetone and mixtures with water enhance the extraction of carotenoids and other lipophilic antioxidants. However, some of these compounds may show reduced reactivity in the ABTS assay due to variations in solubility and compatibility with the medium [66,67].

Antioxidant activity is a vital indicator of a substance’s ability to prevent or reduce oxidative processes. However, these measures are influenced by factors such as the concentration of the sample, the method of quantification used, the specific effect measured, the culture conditions, and the specific part of the plant analyzed [68], as can be seen from the values obtained for the concentration of the antioxidant activity when using the ABTS and DPPH method.

In terms of antioxidant activity, remarkable values were observed in *T. floribunda*, *H. rosa-sinensis*, and *Tibouchina* sp., exhibiting values of 7.80 mmol TE/g DW, 7.67 mmol TE/g DW, and 7.43 mmol TE/g DW, respectively, by ABTS. Conversely, species such as *P. lutea*, *C. argentea*, and *G. nicaraguensis* displayed lower antioxidant activity, with values of 2.5 mmol TE/g DW, 2.47 mmol TE/g DW, and 2.02 mmol TE/g DW. However, *I. hawkeri* (77.77 mmol TE/g DW), *B. americana* (75.87 mmol TE/g DW), *I. walleriana* (73.23 mmol TE/g DW), *A. corymbosa* (67.63 mmol TE/g DW) and *D. pinnata* (65.79 mmol TE/g DW) showed the high antioxidant activity of DPPH. It is important to note that the antioxidant activity of a plant is not solely due to a single compound but results from the synergistic interaction of several bioactive compounds present in the plant [69,70]. These results are essential for the identification of plant candidates with potential applications in the pharmaceutical or cosmetic industry, where natural antioxidants are highly valued for their beneficial effects on human health, and also in the food industry, where phenolic compounds have been used for their antimicrobial and antioxidant activity in food packaging [55].

In this context, the antioxidant activity of *H. rosa-sinensis*, assessed through the DPPH assay, using water, 80% ethanol, and absolute ethanol as solvents at concentrations of 500, 1000, and 2000 mg/L, yielded a range between 2.78% and 80.78% [49], which is comparable to the value reported in this study of 23.33% using methanol. This difference may be due to variations in antioxidant activity caused by different extraction parameters and culture conditions. Similarly, a review on *C. argentea* showed that this species has a marked antioxidant activity [71], which is consistent with the results of this study (51.7%). In addition, a study on edible flowers of different species reported that *H. rosa-sinensis*, grown in areas with temperatures below 25 °C and in varieties of different colours, showed a range of antioxidant activity from 0.44 to 0.77 mmol TE/g DW, values lower than those observed in this work [20]. This suggests that environmental temperature may significantly influence the composition and concentration of antioxidants.

### 4.7. Antimicrobial Activity

Evaluating the flower extracts’ antimicrobial properties included antibacterial and antifungal tests. The bacterial strains *Escherichia coli*, *Staphylococcus aureus*, *Pseudomonas aeruginosa*, and *Streptococcus mutans* and the pathogenic fungi *Candida albicans* and *Candida tropicalis* were tested. These microorganisms are responsible for several human infections, including those of the urinary, respiratory, skin, and oral systems. The ability to prevent their proliferation is of great interest to the healthcare sector, particularly given the increasing resistance to traditional therapeutic approaches [72].

The use of 50% ethanol for extraction and testing of antimicrobial activity provides an optimal balance between extraction efficiency of bioactive compounds and compatibility with the microbiological assay, giving reliable results. This is because a 50% ethanolic solution is sufficiently polar to extract a wide range of phenolic compounds and flavonoids known for their antimicrobial activity [73]. In turn, at high concentrations of ethanol, direct microbial inhibition has been observed, attributed to the solvent itself.

In this context, most of the flower extracts show remarkable antibacterial activity, especially against *S. aureus*, a Gram-positive bacterium widely known for its ability to develop resistance to several antibiotics [74]. Among the most effective extracts were *C. nobilis* (20.5 mm), *P. heteromallum* (18.5 mm), and *A. poiretii* (17.0 mm), which showed significant zones of inhibition. This antimicrobial activity could be attributed to bioactive compounds such as *p*-coumaric acid, caffeic acid, gallic acid, and chlorogenic acid, each of which has been shown to inhibit the growth of *S. aureus* [72,75]. In addition, a study of the methanolic extract of *A. poiretii* flowers confirmed its antibacterial activity against *S. aureus* [76], supporting the potential use of these extracts in developing natural treatments to combat resistant bacterial infections. In turn, a literature review of *C. argentea* suggests that this species has antibacterial activity due to the presence of 4-hydroxybenzoic acid and caffeic acid [71], two compounds present in the species in this study. Another study reported that *Bougainvillea* has activity against *S. aureus*, *E. coli*, *P. aeruginosa*, and other microorganisms [12].

For *E. coli* and *P. aeruginosa*, the results are more heterogeneous. Only a few extracts showed inhibitory activity against *E. coli*, in particular *C. nobilis* (15.5 mm), *M. erythrophylla* (15.0 mm), and *C. spiralis* (13.0 mm). Regarding *P. aeruginosa*, a bacterium notorious for its high antibiotic resistance, some extracts such as *P. heteromallum* (15.0 mm), *C. nobilis* (13.0 mm), and *A. poiretti* (12.0 mm) showed antimicrobial activity. However, most of the extracts did not show significant inhibition against these Gram-negative bacteria, suggesting that the bioactive compounds present in the flowers may have less efficacy against these bacteria’s complex cell wall structure. In this context, previous studies have reported anthelmintic activity of *M. erythophylla* [77] and antimicrobial activity against *S. aureus* and *E. coli* [76], as well as inhibition of *E. coli* by *B. spectabilis* [78,79], possibly due to the presence of isophitol [80]; however, in the present study, the latter species did not show antimicrobial activity against *E. coli*.

*S. mutans*, a key pathogen in the development of dental caries [81], was significantly inhibited by several flower extracts. The most effective extract was *B. spectabilis*, with an inhibition halo of 22.0 mm, followed by *P. heteromallum* (21.0 mm) and *B. macrophylla* (17.0 mm). These results suggest a remarkable antimicrobial potential of these extracts, which could make them suitable for developing products focused on oral health.

On the other hand, the antifungal activity of the extracts studied was limited. Only the extract of *P. heteromallum* showed significant activity against *C. albicans* (13.0 mm), while no extract was effective against *Candida tropicalis*. This suggests that the antifungal compounds present in the flowers are specific in their action and do not have a broad spectrum against different fungal species. In this regard, other studies on *C. argentea* showed antifungal activity against *C. albicans*, as well as antibacterial activity against *S. aureus*, *P. aeruginosa*, *E. coli*, and other microorganisms [71].

These showed superior inhibition zones compared to reference antibiotics, particularly against *S. mutans* (30.0 mm) and *S. aureus* (24.8 mm). Although some flower extracts showed promising results, the data suggest that they may require optimization or synergy with other bioactive compounds to match or exceed the efficacy of conventional antimicrobial treatments.

### 4.8. Statistical Analysis

Principal component analysis included weight (W), longitudinal (DL) and equatorial diameter (DE), pH, soluble solids (SS), total titratable acid (AT), moisture (H), ash (AS), total carotenoids (CT), total phenolics (PT), total anthocyanins (AN), organic acids (OA), antioxidant activity of ABTS (AB), antioxidant activity of DPPH (DP), and inhibition size for *Escherichia coli* (Ec), *Staphylococcus aureus* (Sa), *Pseudomonas aeruginosa* (Pa), *Streptococcus mutans* (Sm), *Candida albicans* (Ca), and *Candida tropicalis* (Ct). Principal component analysis (Figure 5A,B) showed that the highest variance was explained by Dim1 (11.4%), followed by Dim2 (9.6%) when all variables were considered, and when the total sum of bioactive compounds was considered, Dim1 explained 17.1% and Dim2 14.1%. A relationship between the antioxidant activity of ABTS, organic acids, and total phenolics and *E. coli* and *S. aureus* was observed. There was also a relationship between pH and total carotenoids and ash, titratable acidity, antioxidant activity of DPPH, total anthocyanins, and moisture. In addition, Figure 5A showed an inverse relationship between flower size and flower weight with titratable acidity. These relationships agree with the results reported by other authors [29]. On the other hand, the principal component analysis, considering all the study variables, showed that Fuchsia species showed an important contribution of organic compounds, especially malic acid and the contribution of gallic acid.

The correlation analysis included weight (W), longitudinal (DL) and equatorial diameter (DE), pH, soluble solids (SS), total titratable acid (AT), moisture (H), ash (AS), total carotenoids (CT), total phenolics (PT), total anthocyanins (AN), organic acids (OA), antioxidant activity of ABTS (AB), antioxidant activity of DPPH (DP), Gallic acid (Ga), protocatechuic acid (Pr), p-coumaric acid (p-Cu), o-coumaric acid (o-Cu), vanillic acid(Va), syringic acid (Si), chlorogenic acid (Ch), caffeic acid (Cat), 4-hydroxi-benzoic acid (4-Hi), caffeic acid (Ca), ferulic acid (Fe), naringin (Na), rutin (Ru), quercetin (Que), quercetin glucoside (G-Que), kamferol (Kam), PT2, total phenolics, citric acid (Cit), malic acid (Mal), tartaric acid (Tar), and inhibition size for *Escherichia coli* (Ec), *Staphylococcus aureus* (Sa), *Pseudomonas aeruginosa* (Pa), S*treptococcus mutans* (Sm), *Candida albicans* (Ca), and *Candida tropicalis* (Ct).

The correlation analysis in Figure 5C,D revealed several relationships between the studied variables. Thus, Figure 5C shows a positive correlation between weight and size, an association widely documented in plant species. Similarly, the antioxidant activity of the DPPH method was correlated with total anthocyanins, ash with total carotenoids, *S. aureus* with *E. coli* and *P. aeruginosa*. In contrast, soluble solids were inversely correlated with pH and titratable acidity, pH with *S. aureus* and *E. coli*, and total carotenoids with total anthocyanins. These results suggest that smaller fruits tend to have a higher concentration of soluble solids, which could inhibit glycolysis, reduce phenolic content, and increase pH [9].

Moreover, specific correlations were observed between the study variables (Figure 5D), the most representative of which are described below. A positive correlation was observed between organic acids with gallic acid, titratable acids with caffeic acid, antioxidant acids with malic acid, gallic acid with malic acid. A negative correlation was also found between pH and total phenolics and *E. coli, S. aureus, P. aeruginosa, S. mutans*, and *S. aureus* with chlorogenic acid and quercetin glycosides. These results highlight the complex relationship between antimicrobial activity and pH, as the antimicrobial agent variably influences pH. In the dental context, low pH conditions can promote the development of caries [82]. Likewise, pH plays a crucial role in skin health and wound healing, affecting protease activity, bacterial growth, and the efficacy of antibacterial agents, which has important implications for dressing design and diagnosis of pH-sensitive wounds [83].

## 5. Conclusions

Flowers are an important source of bioactive compounds. Certain species studied showed high values; *P. urvilleana* for pH, *P. lutea* for soluble solids, *A. corymbosa* for titratable acidity. Furthermore, extraction optimization showed that the concentration of carotenoids is strongly influenced by the plant matrix, whereas phenolic compounds are influenced by the type of solvent. In this respect, *R. alpinia* showed high concentrations of total carotenoids, *T. sorensis* of total phenolics, *P. heteromallum* of total anthocyanins, *H. rosa-sinensis* of citric acid, *A. piretti* of malic acid, and *C. spiralis* of tartaric acid. In addition, the highest concentrations of individual phenolic compounds were found in *A. poiretti* (*m*-coumaric acid), *B. arborea* (4-hydroxybenzoic acid), and *D. pinnata* (kaempferol). Regarding antioxidant activity, high values were observed in *T. floribunda* using the ABTS method and in *I. hawkeri* using the DPPH method. Finally, species such as *A. poiretti*, *B. macrophylla*, and *C. nobilis* showed inhibitory activity against *Escherichia coli*, *Staphylococcus aureus*, *Pseudomonas aeruginosa*, and *Streptococcus mutans*. In contrast, *P. heteromallum* showed inhibitory activity against *Candida albicans*. These results highlight the complex relationship between the different organic compounds in flower petals and indicate the need for further studies to unravel the potential health benefits.

## Figures and Tables

**Table 1:** Floral species are under study.

N°	Family	Species	Altitude (masl)
1	Acanthaceae	*Megaskepasma erythrochlamys* Lindau	1020
2	Acanthaceae	*Odontonema cuspidatum* (Nees) Kuntze	562
3	Acanthaceae	*Pachystachys lutea* Nees	981
4	Acanthaceae	*Sanchezia oblonga* Ruiz & Pav.	1382
5	Acanthaceae	*Thunbergia grandiflora* Roxb.	1240
6	Amaranthaceae	*Celosia argentea* L.	136
7	Apiaceae	*Eryngium aquaticum* L.	1260
8	Apocynaceae	*Allamanda cathartica* L.	40
9	Araceae	*Philodendron fragrantissimum* (Hook.) G.Don	562
10	Araceae	*Philodendron strictum* G.S. Bunting	562
11	Araceae	*Xanthosoma robustum* Schott	620
12	Asteraceae	*Acmella papposa* (Hemsl.) R.K. Jansen	1486
13	Asteraceae	*Dahlia pinnata* Cav.	2222
14	Asteraceae	*Dendrophorbium balsapampae* (Cuatrec.) B. Nord.	2447
15	Asteraceae	*Pseudogynoxys chenopodioides* (Kunth) Cabrera	1360
16	Balsaminaceae	*Impatiens hancockii* C.H.Wright.	2593
17	Balsaminaceae	*Impatiens hawkeri* W. Bull	630
18	Balsaminaceae	*Impatiens sodenii* Engl. & Warb. ex Engl.	2814
19	Balsaminaceae	*Impatiens walleriana* Hook.f.	630
20	Bromeliaceae	*Aechmea corymbosa* (Mart. Ex Schult. & Schult.f.) Mez	1506
21	Bromeliaceae	*Guzmania nicaraguensis* Mez & Baker	1580
22	Bromeliaceae	*Ronnbergia veitchii* (Baker) Aguirre-Santoro	1337
23	Campanulaceae	*Centropogon cornutus* (L.) Druce	562
24	Caricaceae	*Carica papaya* L.	562
25	Convolvulaceae	*Ipomoea triloba* L.	16
26	Costaceae	*Costus spiralis* (Jacq.) Roscoe	1320
27	Clusiaceae	*Clusia nitida* Bittrich & F.N. Cabral	1477
28	Ericaceae	*Cavendishia bracteata* (Ruiz & Pav. Ex J.St.-Hil.) Hoerold	2100
29	Ericaceae	*Cavendishia cuatrecasasii* A.C.Sm.	1910
30	Ericaceae	*Cavendishia nobilis* Lindl.	2060
31	Ericaceae	*Thibaudia floribunda* Kunth	1641
32	Euphorbiaceae	*Acalypha poiretii* Spreng.	620
33	Fabaceae	*Brownea macrophylla* Linden	940
34	Fabaceae	*Calliandra angustifolia* Spruce ex Benth.	1598
35	Fabaceae	*Erythrina americana* Mill.	562
36	Gentianaceae	*Macrocarpaea sodiroana* Gilg	1490
37	Gesneriacea	*Glossoloma ichthyoderma* (Hanst.) J.L. Clark	1495
38	Heliconiaceae	*Heliconia episcopalis* Vell.	620
39	Heliconiaceae	*Heliconia collinsiana* Griggs	1641
40	Heliconiaceae	*Heliconia latispatha* Benth.	1494
41	Heliconiaceae	*Heliconia rostrata Ruiz & Pav*.	562
42	Heliconiaceae	*Heliconia wagneriana* Petersen	562
43	Lamiaceae	*Prunella vulgaris* L.	1850
44	Malvaceae	*Hibiscus rosa-sinensis* L.	500
45	Marantaceae	*Stromanthe stromanthoides* (J.F. Macbr.) L.Andersson	530
46	Melastomataceae	*Pleroma heteromallum* (D.Don) D. Don	1260
47	Melastomataceae	*Andesanthus lepidotus* (Humb. & Bonpl.) P.J.F.Guim. & Michelang.	1742
48	Melastomataceae	*Chaetogastra mollis* (Bonpl.) DC.	1850
49	Melastomataceae	*Plerona urvilleanum (DC.) PJ:F:Gui. & Michelang*.	1234
50	Musaceae	*Musa velutina* H. Wendl. & Drude	562
51	Nyctaginaceae	*Bougainvillea spectabilis* Willd.	14
52	Orchidaceae	*Elleanthus aurantiacus* (Lindl.) Rchb.f.	2530
53	Orchidaceae	*Oncidium sp.*	306
54	Orchidaceae	*Sobralia liliastrum* Lindl.	1260
55	Rubiaceae	*Mussaenda erythrophylla Schumach. & Thonn*.	510
56	Rubiaceae	*Mussaenda philippica* A. Rich.	940
57	Rubiaceae	*Palicourea angustifolia* Kunth	510
58	Solanaceae	*Brugmansia arborea* (L.) Sweet	1641
59	Solanaceae	*Iochroma gesnerioides* (Kunth) Miers	2054
60	Solanaceae	*Browallia americana* L.	1477
61	Solanaceae	*Brugmansia versicolor* Lagerh.	2833
62	Solanaceae	*Solanum dulcamara L.*	1910
63	Verbenaceae	*Lantana rugulosa* Kunth	2798
64	Zingiberaceae	*Alpinia purpurata* (Vieill.) K. Schum.	14
65	Zingiberaceae	*Hedychium coronarium* J. Koening	940
66	Zingiberaceae	*Hedychium coccineum* Buch.-Ham.exSm.	1240
67	Zingiberaceae	*Renealmia alpinia* (Rottb.) Maas	940

Note: masl, metres above sea level.

**Table 2:** Average values of the physicochemical parameters of the floral species analyzed.

N°	Scientific Name	Weight (g)	DL (cm)	DE (cm)	pH	SS (°Brix)	TA (%)	Humidity (%)	Ash (%)
1	*Megaskepasma erythrochlamys*	0.17 ± 0.03	4.21 ± 0.80	1.36 ± 0.36	9.15 ± 0.24	1.74 ± 0.22	1.44 ± 0.08	85.06 ± 3.32	0.18 ± 0.00
2	*Odontonema cuspidatum*	0.03 ± 0.00	1.69 ± 0.23	0.36 ± 0.08	4.60 ± 0.46	5.04 ± 0.05	0.25 ± 0.00	85.59 ± 0.18	3.53 ± 0.20
3	*Pachystachys lutea*	0.05 ± 0.02	1.77 ± 0.58	1.54 ± 0.82	7.00 ± 0.00	15.80 ± 0.42	1.44 ± 0.19	77.72 ± 4.23	3.46 ± 0.00
4	*Sanchezia oblonga*	4.16 ± 0.37	4.23 ± 1.01	4.16 ± 0.37	7.00 ± 0.00	4.00 ± 0.00	0.48 ± 0.05	81.8 ± 1.22	8.02 ± 0.29
5	*Thunbergia grandiflora*	1.44 ± 0.15	3.99 ± 0.25	7.93 ± 0.74	8.50 ± 0.00	7.00 ± 0.00	0.28 ± 0.02	56.98 ± 4.08	1.85 ± 0.08
6	*Celosia argentea*	21.33 ± 0.92	7.68 ± 0.33	9.52 ± 0.90	6.50 ± 0.00	3.94 ± 0.10	0.12 ± 0.00	80.53 ± 0.25	1.44 ± 0.21
7	*Eryngium aquaticum*	0.10 ± 0.02	1.32 ± 0.22	3.75 ± 1.01	5.50 ± 0.00	10.60 ± 0.97	0.59 ± 0.11	73.77 ± 1.40	2.21 ± 0.13
8	*Allamanda cathartica*	2.29 ± 0.42	9.22 ± 0.24	11.30 ± 0.44	6.00 ± 0.00	3.00 ± 0.00	0.94 ± 0.05	89.55 ± 0.27	0.51 ± 0.04
9	*Philodendron fragrantissimum*	5.72 ± 1.33	8.82 ± 1.25	1.06 ± 0.17	6.00 ± 0.00	0.21 ± 0.07	0.11 ± 0.01	84.35 ± 0.97	3.54 ± 0.29
10	*Philodendron strictum*	6.42 ± 1.92	8.74 ± 2.47	2.54 ± 0.48	4.00 ± 0.00	2.71 ± 0.22	0.19 ± 0.05	91.52 ± 0.34	1.28 ± 0.04
11	*Xanthosoma robustum*	72.79 ± 15.16	23.68 ± 3.11	2.24 ± 0.22	4.30 ± 0.48	3.00 ± 0.00	0.05 ± 0.01	91.77 ± 0.11	1.32 ± 0.01
12	*Acmella papposa*	0.06 ± 0.02	1.28 ± 0.08	1.62 ± 0.24	6.00 ± 0.00	5.14 ± 0.10	1.01 ± 0.15	76.63 ± 0.23	2.39 ± 0.00
13	*Dahlia pinnata*	18.70 ± 3.95	9.96 ± 1.19	9.93 ± 0.93	4.40 ± 0.52	5.40 ± 0.52	0.17 ± 0.06	92.63 ± 0.09	0.67 ± 0.00
14	*Dendrophorbium balsapampae*	0.32 ± 0.03	1.39 ± 0.11	0.89 ± 0.39	6.00 ± 0.00	3.85 ± 0.24	0.34 ± 0.04	76.71 ± 0.05	1.72 ± 0.08
15	*Pseudogynoxys chenopodioides*	0.46 ± 0.17	1.19 ± 0.24	1.48 ± 0.52	5.7 ± 0.48	2.40 ± 0.52	0.32 ± 0.08	73.16 ± 1.17	2.58 ± 0.22
16	*Impatiens hancockii*	1.02 ± 0.00	25.33 ± 3.02	25.33 ± 3.02	4.30 ± 0.48	3.92 ± 0.10	0.75 ± 0.02	93.85 ± 0.36	0.03 ± 0.00
17	*Impatiens hawkeri*	0.22 ± 0.03	5.7 ± 0.45	2.73 ± 0.58	2.40 ± 0.84	3.00 ± 0.00	0.83 ± 0.53	95.74 ± 0.32	0.53 ± 0.01
18	*Impatiens sodenii*	0.91 ± 0.21	3.51 ± 0.19	5.15 ± 0.33	4.00 ± 0.00	3.55 ± 0.44	0.58 ± 0.07	94.02 ± 1.33	0.34 ± 0.08
19	*Impatiens walleriana*	0.22 ± 0.07	1.51 ± 0.63	3.27 ± 1.07	2.00 ± 0.00	3.00 ± 0.00	0.29 ± 0.03	96.05 ± 0.19	0.60 ± 0.02
20	*Aechmea corymbosa*	0.27 ± 0.01	2.62 ± 0.26	0.65 ± 0.06	5.80 ± 0.42	2.00 ± 0.00	3.81 ± 0.45	90.10 ± 0.29	0.25 ± 0.02
21	*Guzmania nicaraguensis*	13.84 ± 2.41	10.30 ± 0.98	2.42 ± 0.26	7.00 ± 0.00	2.85 ± 0.24	0.27 ± 0.07	86.67 ± 0.08	0.98 ± 0.10
22	*Ronnbergia veitchii*	2.18 ± 0.63	5.31 ± 0.53	1.46 ± 0.17	6.60 ± 0.52	2.00 ± 0.00	0.62 ± 0.18	78.51 ± 2.15	0.81 ± 0.05
23	*Centropogon cornutus*	0.47 ± 0.08	4.80 ± 1.01	0.78 ± 0.12	5.00 ± 0.00	8.88 ± 0.27	0.29 ± 0.03	83.40 ± 0.14	0.15 ± 0.01
24	*Carica papaya*	0.33 ± 0.53	1.76 ± 0.81	0.38 ± 0.17	6.00 ± 0.00	1.00 ± 0.00	0.15 ± 0.01	87.73 ± 0.07	0.70 ± 0.37
25	*Ipomoea triloba*	0.17 ± 0.03	4.37 ± 0.48	1.83 ± 0.28	5.45 ± 0.44	10.06 ± 0.10	2.01 ± 0.31	90.12 ± 1.44	0.10 ± 0.01
26	*Costus spiralis*	13.37 ± 4.69	6.60 ± 1.30	2.45 ± 0.40	3.00 ± 0.00	3.70 ± 0.48	0.14 ± 0.01	66.10 ± 5.02	1.30 ± 0.09
27	*Clusia nitida*	7.23 ± 0.93	2.20 ± 0.34	3.45 ± 0.28	4.87 ± 0.02	4.08 ± 0.10	0.49 ± 0.02	90.52 ± 0.85	0.21 ± 0.00
28	*Cavendishia bracteata*	1.60 ± 0.15	4.46 ± 0.20	0.99 ± 0.11	2.73 ± 0.41	4.75 ± 0.18	0.27 ± 0.02	92.30 ± 0.31	0.83 ± 0.02
29	*Cavendishia cuatrecasasii*	0.61 ± 0.07	3.34 ± 0.16	0.52 ± 0.10	3.00 ± 0.00	4.56 ± 1.32	0.99 ± 0.09	92.28 ± 0.12	0.54 ± 0.01
30	*Cavendishia nobilis*	0.73 ± 0.14	3.27 ± 0.36	0.84 ± 0.12	4.00 ± 0.00	4.75 ± 0.41	0.20 ± 0.02	87.04 ± 0.06	0.81 ± 0.05
31	*Thibaudia floribunda*	0.16 ± 0.04	1.67 ± 0.19	0.47 ± 0.11	4.00 ± 0.00	2.53 ± 0.21	1.53 ± 0.18	91.10 ± 0.21	0.47 ± 0.00
32	*Acalypha poiretii*	2.08 ± 1.07	16.70 ± 7.39	0.70 ± 0.17	2.00 ± 0.00	6.10 ± 0.88	1.19 ± 0.09	75.26 ± 0.70	2.19 ± 0.14
33	*Brownea macrophylla*	2.38 ± 0.50	13.45 ± 2.88	32.48 ± 8.65	2.00 ± 0.94	12.00 ± 1.33	0.23 ± 0.04	84.17 ± 0.12	0.70 ± 0.07
34	*Calliandra angustifolia*	0.69 ± 0.12	3.32 ± 0.52	1.74 ± 0.33	3.45 ± 0.44	6.08 ± 0.29	1.15 ± 0.45	80.87 ± 1.64	0.75 ± 0.06
35	*Erythrina americana*	1.07 ± 0.17	8.10 ± 0.33	0.98 ± 0.16	6.00 ± 0.00	2.00 ± 0.00	0.36 ± 0.01	78.69 ± 0.89	0.87 ± 0.05
36	*Macrocarpaea sodiroana*	0.07 ± 0.02	4.00 ± 0.00	1.28 ± 0.55	2.18 ± 0.52	6.00 ± 0.00	2.00 ± 0.00	14.26 ± 1.48	0.46 ± 0.12
37	*Glossoloma ichthyoderma*	0.72 ± 0.28	3.38 ± 0.51	0.97 ± 0.19	5.00 ± 0.00	0.48 ± 0.23	0.25 ± 0.15	81.34 ± 0.54	1.81 ± 0.03
38	*Heliconia episcopalis*	2.18 ± 0.63	5.31 ± 0.53	1.46 ± 0.17	5.60 ± 0.39	3.68 ± 0.54	0.05 ± 0.01	84.74 ± 2.13	2.89 ± 0.15
39	*Heliconia collinsiana*	19.93 ± 2.42	13.43 ± 0.21	3.61 ± 0.30	4.45 ± 0.44	2.86 ± 0.05	0.53 ± 0.09	70.34 ± 0.74	0.66 ± 0.03
40	*Heliconia latispatha*	3.25 ± 0.62	11.20 ± 1.95	1.99 ± 0.12	8.00 ± 0.00	3.00 ± 0.00	0.37 ± 0.08	82.65 ± 0.99	3.11 ± 0.22
41	*Heliconia rostrata*	11.36 ± 2.46	8.51 ± 0.26	3.44 ± 0.18	6.00 ± 0.00	1.00 ± 0.00	0.21 ± 0.03	70.52 ± 0.56	3.98 ± 0.27
42	*Heliconia wagneriana*	29.04 ± 1.37	12.38 ± 0.69	7.78 ± 0.36	7.00 ± 0.00	1.04 ± 0.15	0.22 ± 0.04	85.64 ± 2.89	0.86 ± 0.03
43	*Prunella vulgaris*	0.01 ± 0.00	1.42 ± 0.11	0.36 ± 0.07	7.80 ± 0.26	6.70 ± 0.48	0.51 ± 0.01	84.43 ± 1.28	2.31 ± 0.01
44	*Hibiscus rosa-sinensis*	1.01 ± 0.00	24.43 ± 1.21	5.45 ± 0.91	5.00 ± 0.82	3.20 ± 0.26	0.28 ± 0.02	91.66 ± 0.52	0.35 ± 0.00
45	*Stromanthe stromanthoides*	0.19 ± 0.03	1.61 ± 0.21	0.45 ± 0.08	5.00 ± 0.00	4.60 ± 0.52	0.21 ± 0.01	76.92 ± 3.80	2.09 ± 0.06
46	*Pleroma heteromallum*	0.17 ± 0.23	1.65 ± 0.31	0.47 ± 0.06	6.00 ± 0.00	6.00 ± 0.00	2.73 ± 0.50	86.16 ± 0.50	1.02 ± 0.01
47	*Andesanthus lepidotus*	0.45 ± 0.09	5.12 ± 0.56	5.32 ± 0.69	2.45 ± 0.44	4.01 ± 0.09	1.10 ± 0.04	93.96 ± 0.00	0.33 ± 0.01
48	*Chaetogastra molis*	0.07 ± 0.01	2.08 ± 0.20	1.60 ± 0.41	3.00 ± 0.00	4.80 ± 0.42	2.8 ± 0.75	86.82 ± 0.05	2.53 ± 0.03
49	*Plerona urvilleanum*	0.46 ± 0.05	2.03 ± 0.32	5.18 ± 0.59	11.4 ± 0.52	9.20 ± 1.03	0.30 ± 0.08	88.21 ± 1.03	1.20 ± 0.04
50	*Musa velutina*	39.73 ± 6.53	9.89 ± 0.22	4.31 ± 0.41	4.30 ± 0.48	2.46 ± 0.05	1.03 ± 0.10	91.66 ± 0.52	1.13 ± 0.12
51	*Bougainvillea spectabilis*	0.36 ± 0.08	3.61 ± 0.44	3.06 ± 0.42	4.00 ± 0.00	3.00 ± 0.00	0.41 ± 0.02	83.57 ± 0.47	2.11 ± 0.24
52	*Elleanthus aurantiacus*	0.10 ± 0.03	1.37 ± 0.12	0.58 ± 0.10	5.50 ± 0.75	6.10 ± 0.32	0.43 ± 0.04	90.5 ± 1.69	1.60 ± 0.25
53	*Oncidium sp.*	1.29 ± 0.40	2.82 ± 0.53	5.65 ± 0.38	2.70 ± 0.48	11.20 ± 1.32	0.39 ± 0.02	89.80 ± 0.56	0.39 ± 0.07
54	*Sobralia liliastrum*	6.62 ± 2.23	12.09 ± 1.77	2.09 ± 0.34	5.50 ± 0.41	4.58 ± 1.46	0.14 ± 0.02	92.70 ± 0.14	0.56 ± 0.02
55	*Mussaenda erythrophylla*	0.61 ± 0.16	5.64 ± 0.43	7.89 ± 1.32	5.60 ± 0.52	5.00 ± 1.05	0.29 ± 0.02	77.96 ± 1.92	3.35 ± 0.19
56	*Mussaenda philippica*	0.39 ± 0.18	4.25 ± 1.21	2.93 ± 0.53	6.40 ± 0.52	11.00 ± 0.67	0.61 ± 0.06	78.56 ± 0.69	1.36 ± 0.09
57	*Palicourea angustifolia*	0.24 ± 0.04	2.52 ± 0.27	0.52 ± 0.05	5.60 ± 0.21	4.00 ± 0.27	0.24 ± 0.03	87.85 ± 0.35	1.20 ± 0.01
58	*Brugmansia arborea*	11.54 ± 2.67	22.93 ± 3.84	4.09 ± 0.36	5.23 ± 0.56	6.62 ± 1.27	0.60 ± 0.04	87.34 ± 5.40	0.49 ± 0.05
59	*Iochroma gesnerioides*	0.30 ± 0.05	3.14 ± 0.30	0.45 ± 0.04	5.00 ± 0.00	5.41 ± 0.41	2.68 ± 0.12	86.14 ± 0.15	0.30 ± 0.03
60	*Browallia americana*	0.02 ± 0.00	1.67 ± 0.07	1.35 ± 0.26	6.45 ± 0.44	4.00 ± 0.00	0.31 ± 0.01	87.39 ± 0.69	0.95 ± 0.06
61	*Brugmansia versicolor*	10.91 ± 2.52	17.66 ± 2.99	8.76 ± 0.96	6.40 ± 0.52	7.00 ± 0.00	0.60 ± 0.04	88.96 ± 0.45	0.85 ± 0.02
62	*Solanum dulcamara*	0.10 ± 0.02	2.20 ± 0.36	1.78 ± 0.51	10.10 ± 0.77	3.68 ± 0.99	1.24 ± 0.25	91.00 ± 0.32	0.98 ± 0.03
63	*Lantana rugulosa*	0.01 ± 0.00	1.07 ± 0.11	1.02 ± 0.09	7.00 ± 0.00	5.46 ± 0.47	0.20 ± 0.01	70.49 ± 0.05	3.35 ± 0.25
64	*Alpinia purpurata*	28.97 ± 4.45	23.75 ± 2.06	62.85 ± 14.6	3.00 ± 0.00	1.40 ± 0.52	0.15 ± 0.03	87.88 ± 2.76	4.02 ± 0.14
65	*Hedychium coronarium*	2.31 ± 0.66	10.92 ± 1.78	13.08 ± 5.38	6.60 ± 0.21	2.64 ± 0.31	0.47 ± 0.04	94.70 ± 0.11	1.09 ± 0.01
66	*Hedychium coccineum*	0.23 ± 0.03	4.29 ± 0.61	1.73 ± 0.32	7.00 ± 0.00	5.00 ± 0.00	1.06 ± 0.09	42.04 ± 4.81	6.12 ± 1.32
67	*Renealmia alpinia*	0.24 ± 0.04	2.52 ± 0.27	0.52 ± 0.05	5.60 ± 0.21	4.00 ± 0.27	0.24 ± 0.03	53.70 ± 1.70	1.20 ± 0.01

Note: DL, longitudinal diameter; DE, equatorial diameter; SS, soluble solid; TA, total titratable acidity.

**Table 3:** Average values of carotenoids, phenolics, and antioxidant activity concentration of the floral species analyzed.

N°	Scientific Name	Total Carotenoids (mg β-Carotene/g DW)	Total Phenolics (mg GAE/g DW)	Total Anthocyanins (mg C-3-gl/g DW)	Citric Acid (mg/100 g DW)	Malic Acid (mg/100 g DW)	Tartaric Acid (mg/100 g DW)	Antioxidant Activity (mmol TE/g) ABTS	Antioxidant Activity (mmol TE/g) DPPH
1	*M. erythrochlamys*	1.53 ± 0.02	76.09 ± 1.01	0.28 ± 0.03	116.4 ± 12.3	341.6 ± 5.2	213.4 ± 8.0	6.04 ± 0.70	31.89 ± 2.79
2	*O. cuspidatum*	3.04 ± 0.00	223.24 ± 4.66	0.20 ± 0.04	320.9 ± 7.5	141.7 ± 3.1	59.2 ± 3.4	5.96 ± 1.04	20.76 ± 0.57
3	*P. lutea*	83.03 ± 8.81	104.41 ± 0.75	0.13 ± 0.02	142.6 ± 1.0	112.8 ± 3.8	54.8 ± 1.2	2.50 ± 0.40	23.42 ± 3.49
4	*S. oblonga*	2.95 ± 0.02	194.55 ± 6.28	0.16 ± 0.01	42.9 ± 3.5	322.8 ± 10.9	344.4 ± 5.9	4.63 ± 0.02	31.50 ± 2.89
5	*T. grandiflora*	0.82 ± 0.02	110.25 ± 0.54	0.16 ± 0.01	20.2 ± 1.8	74.0 ± 1.3	106.6 ± 6.6	5.22 ± 0.04	48.42 ± 3.75
6	*C. argentea*	1.33 ± 0.01	140.63 ± 0.85	0.10 ± 0.02	51.6 ± 3.3	122.3 ± 10.6	63.6 ± 1.2	2.47 ± 0.57	28.00 ± 0.67
7	*E. aquaticum*	15.04 ± 0.56	85.81 ± 2.03	0.36 ± 0.08	84.8 ± 1.5	4605.0 ± 308.3	103.0 ± 3.9	4.96 ± 0.07	32.26 ± 3.90
8	*A. cathartica*	32.53 ± 0.09	167.12 ± 3.72	0.16 ± 0.01	613.6 ± 19.9	261.3 ± 21.3	106.7 ± 0.0	3.70 ± 0.85	25.73 ± 2.07
9	*P. fragrantissimum*	1.80 ± 0.02	291.75 ± 10.84	0.59 ± 0.08	44.1 ± 0.9	249.1 ± 1.3	22.0 ± 0.3	6.09 ± 0.92	33.41 ± 1.14
10	*P. strictum*	1.81 ± 0.05	353.57 ± 17.01	0.72 ± 0.15	923.3 ± 64.9	241.3 ± 7.1	13.6 ± 0.3	4.84 ± 0.60	46.05 ± 2.11
11	*X. robustum*	0.59 ± 0.01	94.45 ± 3.11	0.50 ± 0.16	391.9 ± 3.5	37.8 ± 0.9	34.3 ± 1.0	5.05 ± 0.03	47.75 ± 1.88
12	*A. papposa*	211.00 ± 0.57	92.39 ± 1.21	0.31 ± 0.06	112.7 ± 6.6	262.6 ± 43.8	104.8 ± 0.3	3.96 ± 0.02	36.53 ± 4.46
13	*D. pinnata*	1.11 ± 0.03	199.65 ± 2.50	1.84 ± 0.19	106.8 ± 23.7	1735.6 ± 275.3	92.7 ± 15.2	4.46 ± 0.20	65.79 ± 7.14
14	*D. balsapampae*	129.43 ± 12.75	133.07 ± 2.89	2.04 ± 0.12	168.5 ± 18.9	156.9 ± 10.2	226.9 ± 2.5	5.86 ± 0.08	64.46 ± 9.82
15	*P. chenopodioides*	1.87 ± 0.01	114.09 ± 4.62	0.31 ± 0.03	206.5 ± 2.8	431.4 ± 10.8	316.2 ± 5.8	4.55 ± 0.04	21.02 ± 0.87
16	*I. hancockii*	2.07 ± 0.11	165.15 ± 18.36	0.40 ± 0.01	39.1 ± 3.5	30.3 ± 0.0	35.7 ± 1.2	7.33 ± 0.10	32.73 ± 5.26
17	*I. hawkeri*	18.82 ± 0.65	215.17 ± 2.00	0.69 ± 0.09	105.9 ± 4.6	360.5 ± 20.3	222.9 ± 17.2	5.04 ± 0.03	77.77 ± 5.82
18	*I. sodenii*	23.86 ± 2.34	253.43 ± 11.18	0.30 ± 0.02	125.8 ± 9.2	95.5 ± 0.7	116.8 ± 2.3	4.07 ± 0.15	54.92 ± 6.94
19	*I. walleriana*	19.83 ± 1.83	288.17 ± 4.94	0.77 ± 0.03	76.7 ± 1.7	229.0 ± 2.6	129.5 ± 1.6	4.22 ± 1.00	73.23 ± 6.44
20	*A. corymbosa*	12.95 ± 0.14	302.67 ± 14.21	0.50 ± 0.05	62.4 ± 4.7	298.4 ± 1.8	31.3 ± 1.2	4.59 ± 0.04	67.63 ± 2.15
21	*G. nicaraguensis*	3.97 ± 0.06	87.75 ± 1.11	0.20 ± 0.02	254.3 ± 2.6	301.3 ± 28.6	48.7 ± 0.0	2.02 ± 0.08	32.61 ± 5.38
22	*R. veitchii*	1.35 ± 0.01	72.62 ± 0.49	0.04 ± 0.00	272.1 ± 3.1	979.4 ± 16.4	836.6 ± 16.4	4.00 ± 0.06	16.29 ± 1.00
23	*C. cornutus*	1.11 ± 0.01	262.11 ± 15.33	0.23 ± 0.04	956.7 ± 34.6	11655.9 ± 251.3	75.6 ± 1.6	2.86 ± 0.46	34.61 ± 2.44
24	*C. papaya*	2.43 ± 0.02	223.18 ± 7.29	0.23 ± 0.03	435.0 ± 10.6	490.9 ± 12.3	313.7 ± 13.2	5.02 ± 0.26	27.81 ± 2.79
25	*I. triloba*	0.62 ± 0.08	157.49 ± 14.03	0.37 ± 0.09	1333.2 ± 89.2	651.9 ± 2.1	30.5 ± 0.1	5.74 ± 0.05	23.96 ± 4.85
26	*C. spiralis*	0.69 ± 0.04	121.13 ± 0.44	0.76 ± 0.06	137.7 ± 10.7	109.8 ± 5.2	4.8 ± 0.3	5.26 ± 0.01	18.48 ± 1.44
27	*C. nitida*	1.18 ± 0.01	292.34 ± 8.24	0.91 ± 0.05	713.8 ± 76.6	225.5 ± 11.7	170.7 ± 23.4	3.88 ± 0.90	33.25 ± 3.28
28	*C. bracteata*	1.00 ± 0.09	232.83 ± 6.52	0.08 ± 0.00	443.7 ± 1.5	186.7 ± 3.4	143.4 ± 9.5	5.03 ± 0.02	25.84 ± 4.21
29	*C. cuatrecasasii*	0.25 ± 0.02	108.28 ± 6.40	0.16 ± 0.00	48.7 ± 7.8	1002.6 ± 5.8	188.6 ± 20.3	5.02 ± 0.03	40.99 ± 3.91
30	*C. nobilis*	0.73 ± 0.04	151.12 ± 4.14	0.03 ± 0.00	260.8 ± 18.6	278.3 ± 12.0	157.7 ± 29.7	5.01 ± 0.07	17.89 ± 2.61
31	*T. floribunda*	1.39 ± 0.03	259.37 ± 3.35	0.29 ± 0.06	3516.1 ± 154.1	209.4 ± 18.9	87.8 ± 10.2	7.80 ± 0.06	52.46 ± 3.96
32	*A. poiretii*	2.54 ± 0.06	269.57 ± 2.06	0.73 ± 0.06	205.4 ± 19.6	15613.6 ± 1238.4	55.2 ± 4.6	2.95 ± 0.47	44.11 ± 5.50
33	*B. macrophylla*	1.58 ± 0.05	299.60 ± 4.87	2.44 ± 0.38	114.3 ± 0.8	85.9 ± 1.3	51.8 ± 0.3	5.06 ± 0.02	56.74 ± 7.54
34	*C. angustifolia*	2.18 ± 0.05	337.21 ± 3.78	0.17 ± 0.01	158.8 ± 10.3	123.5 ± 12.8	196.0 ± 6.2	4.68 ± 0.03	49.59 ± 1.38
35	*E. americana*	0.84 ± 0.04	258.43 ± 14.93	0.35 ± 0.05	2874.9 ± 101.0	656.1 ± 13.8	439.3 ± 13.6	4.31 ± 1.00	39.36 ± 3.48
36	*M. sodiroana*	1.41 ± 0.16	175.38 ± 36.04	0.88 ± 0.13	142.3 ± 0.5	60.7 ± 1.2	6.9 ± 0.2	3.31 ± 0.48	16.74 ± 1.50
37	*G. ichthyoderma*	0.66 ± 0.06	251.00 ± 13.57	0.37 ± 0.04	28.0 ± 0.4	186.5 ± 1.9	334.7 ± 2.4	3.86 ± 0.89	47.84 ± 2.16
38	*H. episcopalis*	1.55 ± 0.13	56.86 ± 0.50	0.09 ± 0.00	346.9 ± 6.0	761.3 ± 12.6	17.4 ± 0.3	3.02 ± 0.03	29.28 ± 3.04
39	*H. collinsiana*	1.78 ± 0.01	300.94 ± 13.94	0.15 ± 0.04	92.1 ± 7.3	1553.1 ± 8.4	104.1 ± 0.8	4.57 ± 0.02	24.92 ± 3.65
40	*H. latispatha*	0.43 ± 0.01	86.40 ± 1.09	0.11 ± 0.06	45.0 ± 10.0	114.6 ± 0.5	5488.2 ± 70.6	4.65 ± 0.02	15.98 ± 0.70
41	*H. rostrata*	2.49 ± 0.08	155.21 ± 9.89	0.57 ± 0.01	164.9 ± 14.1	637.7 ± 4.1	357.0 ± 75.7	2.56 ± 0.26	22.53 ± 1.77
42	*H. wagneriana*	2.43 ± 0.00	288.17 ± 4.94	0.18 ± 0.04	3800.5 ± 104.4	1327.4 ± 40.0	61.0 ± 1.1	4.22 ± 1.00	46.45 ± 3.57
43	*P. vulgaris*	1.92 ± 0.05	110.69 ± 10.96	0.34 ± 0.06	90.5 ± 0.2	141.8 ± 14.7	25.0 ± 3.1	4.99 ± 0.04	59.43 ± 6.32
44	*H. rosa-sinensis*	3.64 ± 0.13	290.30 ± 9.67	1.93 ± 0.45	17818.6 ± 475.5	1693.8 ± 48.2	46.1 ± 7.2	7.67 ± 0.12	62.15 ± 1.08
45	*S. stromanthoides*	2.64 ± 0.00	50.60 ± 1.55	0.18 ± 0.03	39.6 ± 5.6	55.5 ± 6.8	5.6 ± 1.9	2.63 ± 0.08	24.73 ± 3.20
46	*P. heteromallum*	0.93 ± 0.05	344.22 ± 2.63	7.35 ± 0.43	88.5 ± 7.9	2254.1 ± 33.6	51.3 ± 0.9	4.18 ± 0.95	56.30 ± 2.91
47	*A. lepidotus*	1.42 ± 0.04	246.19 ± 16.01	0.29 ± 0.05	56.1 ± 0.7	5591.2 ± 64.0	137.4 ± 9.8	7.43 ± 0.13	31.76 ± 4.02
48	*C. mollis*	0.86 ± 0.07	354.20 ± 1.20	1.67 ± 0.08	51.1 ± 3.3	161.5 ± 6.8	708.2 ± 32.6	5.01 ± 0.07	55.69 ± 5.02
49	*P. urvilleana*	1.00 ± 0.04	510.53 ± 34.59	1.01 ± 0.01	55.2 ± 13.4	3540.8 ± 173.8	255.7 ± 25.7	4.66 ± 0.03	53.66 ± 4.49
50	*M. velutina*	0.92 ± 0.04	219.08 ± 8.51	0.42 ± 0.01	105.3 ± 10.6	846.2 ± 62.5	39.6 ± 8.1	6.58 ± 0.08	26.27 ± 3.13
51	*B. spectabilis*	2.65 ± 0.02	327.01 ± 8.42	0.06 ± 0.00	175.2 ± 12.5	394.0 ± 4.4	3197.1 ± 401.1	5.65 ± 0.07	29.85 ± 2.64
52	*E. aurantiacus*	1.12 ± 0.08	74.77 ± 2.06	0.13 ± 0.02	59.6 ± 7.2	293.1 ± 2.8	154.2 ± 11.4	4.98 ± 0.04	28.42 ± 3.65
53	*Oncidium sp.*	13.76 ± 0.12	99.11 ± 4.48	0.75 ± 0.06	201.0 ± 9.2	216.0 ± 1.8	171.3 ± 12.0	5.01 ± 0.08	26.56 ± 1.05
54	*S. liliastrum*	0.62 ± 0.01	97.89 ± 3.01	0.14 ± 0.03	365.5 ± 8.0	735.3 ± 56.3	30.6 ± 0.1	3.37 ± 0.11	38.61 ± 5.56
55	*M. erythrophylla*	0.92 ± 0.11	153.47 ± 3.39	0.74 ± 0.06	171.8 ± 5.7	740.0 ± 16.4	350.4 ± 14.9	5.11 ± 0.03	55.21 ± 9.54
56	*M. philippica*	1.99 ± 0.05	202.06 ± 7.07	0.17 ± 0.03	24.9 ± 1.6	87.3 ± 7.6	7.5 ± 0.58	5.03 ± 0.03	25.33 ± 5.74
57	*P. angustifolia*	0.84 ± 0.09	79.42 ± 1.43	0.21 ± 0.05	126.2 ± 9.6	3121.3 ± 260.7	92.5 ± 7.6	2.54 ± 0.04	18.64 ± 1.46
58	*B. arborea*	0.89 ± 0.04	153.37 ± 16.54	0.16 ± 0.05	878.1 ± 25.9	13.2 ± 0.5	197.1 ± 6.1	4.29 ± 0.03	27.70 ± 3.26
59	*I. gesnerioides*	1.15 ± 0.03	273.93 ± 5.84	0.47 ± 0.05	2662.8 ± 1581	666.3 ± 8.0	276.8 ± 9.7	5.74 ± 0.02	30.59 ± 6.11
60	*B. americana*	2.01 ± 0.02	165.91 ± 19.98	0.16 ± 0.01	782.6 ± 54.2	483.3 ± 22.8	1519.5 ± 33.5	4.40 ± 0.02	75.87 ± 4.75
61	*B. versicolor*	3.10 ± 0.16	120.74 ± 9.98	0.13 ± 0.04	1366.5 ± 67.7	764.5 ± 29.9	116.3 ± 0.1	4.70 ± 0.37	52.89 ± 4.57
62	*S. dulcamara*	3.65 ± 0.02	110.94 ± 7.18	0.40 ± 0.01	498.5 ± 34.6	182.1 ± 7.9	67.3 ± 0.2	4.96 ± 0.03	19.35 ± 4.53
63	*L. rugulosa*	0.89 ± 0.05	268.80 ± 13.73	0.31 ± 0.01	195.4 ± 15.4	1152.5 ± 13.5	34.0 ± 0.09	4.78 ± 1.13	40.97 ± 2.21
64	*A. purpurata*	1.33 ± 0.04	344.68 ± 5.80	1.32 ± 0.08	58.1 ± 0.4	189.5 ± 9.6	38.6 ± 1.0	5.72 ± 0.06	37.98 ± 3.63
65	*H. coronarium*	1.19 ± 0.00	75.57 ± 1.11	0.52 ± 0.07	214.9 ± 13.8	89.1 ± 2.9	16.0 ± 1.2	3.78 ± 0.05	30.00 ± 4.07
66	*H. coccineum*	1.71 ± 0.10	86.60 ± 0.81	0.23 ± 0.04	245.2 ± 1.51	100.7 ± 4.9	14.9 ± 0.6	4.90 ± 0.20	24.57 ± 1.36
67	*R. alpinia*	292.50 ± 0.89	34.50 ± 0.77	0.29 ± 0.00	142.1 ± 1.10	98.3 ± 3.2	9.2 ± 0.6	4.85 ± 0.20	42.11 ± 2.78

**Table 4:** Average values of individual phenolics (mg/100 g DW) of the floral species analyzed.

N°	Scientific Name	Gallic Acid	Protocatechuic Acid	*p*-Coumaric Acid	*m*-Coumaric Acid	Syringic Acid	Chlorogenic Acid	4-Hydroxibenzoic Acid	Caffeic Acid	Ferulic Acid	Rutin	Quercetin	Quercetin Glucoside	Kamferol	Total
1	*M. erythrochlamys*	41.1 ± 1.3				778.8 ± 7.6			725.3 ± 53.8	2196.2 ± 39.8	135.0 ± 6.1	450.0 ± 3.1	118.4 ± 9.6		4444.8 ± 89.8
2	*O. cuspidatum*	24.0 ± 1.0		349.1 ± 8.4					2536.1 ± 11.7	1957.5 ± 34.9			712.1 ± 16.5		5578.8 ± 72.4
3	*P. lutea*	92.3 ± 4.1		117.8 ± 1.3		46.8 ± 4.5		666.3 ± 41.2			225.5 ± 3.4		235.8 ± 4.4		1384.6 ± 39.6
4	*S. oblonga*	17.6 ± 1.2		159.4 ± 1.3			60.6 ± 1.5	17.6 ± 1.1	820.8 ± 60.5	2605.1 ± 48.5	33.6 ± 0.4				3714.7 ± 108.9
5	*T. grandiflora*	212.6 ± 2.1			302.2 ± 6.0			106.5 ± 0.2	509.9 ± 35.9	5848.8 ± 8.6	814.8 ± 4.5				7795.2 ± 36.1
6	*C. argentea*	81.8 ± 2.7				41.5 ± 1.2		164.7 ± 3.7	242 ± 30.1		67.2 ± 0.5		431.2 ± 11.7	89.6 ± 3.2	1118.0 ± 45.2
7	*E. aquaticum*	13.2 ± 0.6	43.3 ± 1.7				621 ± 4.9		227.5 ± 1.0				602.6 ± 22.9		1507.6 ± 31.1
8	*A. cathartica*	21.1 ± 2.0		503.9 ± 6.1	219.9 ± 13.9		66.3 ± 5.0	133.6 ± 12.7	307.7 ± 31.5	637 ± 32.9	140.4 ± 23.2		138.9 ± 13.5		2168.6 ± 128.7
9*	*P. fragrantissimum*	15.5 ± 0.6				22.5 ± 3.4		148.7 ± 11.8							655.0 ± 7.1
10	*P. strictum*	78.4 ± 4.1									427.3 ± 0.4	793.8 ± 1.1			1299.5 ± 4.8
11	*X. robustum*	46.2 ± 1.3	36.3 ± 0.2				224.7 ± 0.8	141.6 ± 22.2			380.6 ± 12.9		152.1 ± 4.2		981.6 ± 30.1
12*	*A. papposa*	24.3 ± 0.1		164.2 ± 1.3	165.0 ± 6.5			128.1 ± 2.7	1514.7 ± 56.8	204.8 ± 22.3					2208.3 ± 86.9
13	*D. pinnata*	114.1 ± 0.1				122.7 ± 2.7					601.3 ± 23	5118.6 ± 23.8	1047.4 ± 161.9	8235.5 ± 214.8	15239.6 ± 8.9
14*	*D. balsapampae*						314.3 ± 6.6	308.8 ± 4.8			657.5 ± 1.3				2712.3 ± 66
15	*P. chenopodioides*	37.2 ± 2.2		104.0 ± 2.2				118.7 ± 0.8			207.1 ± 4.8		159.1 ± 10.3		626.0 ± 6.3
16*	*I. hancockii*	14.5 ± 0.4	28.6 ± 0.5		23.5 ± 0.5			133.5 ± 6.8	108.6 ± 15.0	148.2 ± 7.5	152 ± 1.3		117.1 ± 0.9	117.8 ± 0.3	851.1 ± 26.7
17	*I. hawkeri*	42.5 ± 1.3	42.7 ± 0.4	632.3 ± 0.8			104.4 ± 1.5	185.9 ± 7.8	2202.9 ± 32.7					100.0 ± 4.6	3310.7 ± 5.5
18	*I. sodenii*	32.7 ± 2.9	55 ± 4.2								378.5 ± 17.8	450.0 ± 3.1	2405 ± 115.7	577.5 ± 25.2	3448.7 ± 165.2
19	*I. walleriana*	53.6 ± 5.3	59.6 ± 0.6	613.0 ± 14.4			126.4 ± 2.2	183.9 ± 6.1	2838.4 ± 42.5					164.6 ± 2.0	4039.4 ± 17.2
20	*A. corymbosa*	52.3 ± 2.0				25.6 ± 0.3		138.3 ± 4.2	5892.5 ± 172.2	757 ± 2	123.7 ± 13.1				6989.3 ± 159.1
21	*G. nicaraguensis*	30.5 ± 1.1		186.1 ± 4.6				736.5 ± 46.9	1443.6 ± 7.0	557.8 ± 56.2					2954.5 ± 113.6
22*	*R. veitchii*	89.6 ± 1.5		100.9 ± 8.5			477.2 ± 0.7	333.2 ± 8.8			258.2 ± 0.3		176.0 ± 10.5		1460.9 ± 12.7
23	*C. cornutus*	869.4 ± 2.0		1267.6 ± 96.0					329 ± 4.4		168.4 ± 0.3		2013.0 ± 2.8		4647.4 ± 99.3
24	*C. papaya*	80.6 ± 3.1							4005 ± 113.3		810.2 ± 1.5				4895.8 ± 108.7
25	*I. triloba*	117.3 ± 3.4						304.6 ± 3.0	947.5 ± 99.3				32.8 ± 3.0		1402.2 ± 102.7
26*	*C. spiralis*	32.9 ± 1.1				314.3 ± 1.8		147.1 ± 13.2					59.8 ± 3.2		583.4 ± 16.3
27*	*C. nitida*	27.4 ± 0.0	38.7 ± 2.1				356 ± 24.3				55.3 ± 0.0		59.1 ± 3.4		1449.2 ± 60.7
28	*C. bracteata*			2928.5 ± 97.2			240.2 ± 9.2		291.2 ± 10.6						3459.9 ± 117
29*	*C. cuatrecasasii*		27.2 ± 1.1	1170.4 ± 34.0			77.5 ± 2.1				91.4 ± 4.5		192.3 ± 6.5	46.7 ± 6.0	1640.4 ± 12.3
30	*C. nobilis*			2149.5 ± 14.8			158.8 ± 2.1		204 ± 8.8						2512.2 ± 35.8
31*	*T. floribunda*	10.4 ± 0.2		252.4 ± 18.5					1133 ± 63.5	552 ± 30.6					1960.3 ± 112.5
32	*A. poiretii*	81.5 ± 0.7			12,044.3 ± 58.7	3224.7 ± 9.5									15,350.2 ± 35.6
33	*B. macrophylla*	4.3 ± 0.1	133.2 ± 1.0	345.8 ± 9.7		439.3 ± 4.4		65.7 ± 1.2			59.1 ± 1.1		106.1 ± 1.7	41.5 ± 1.4	1194.9 ± 18.7
34	*C. angustifolia*	51.4 ± 3.4			1960.5 ± 1.2		709.4 ± 12.3	113.2 ± 0.9			3814.4 ± 10.1		175.6 ± 1.6		6824.5 ± 29.5
35	*E. americana*	92.4 ± 6.2				402.9 ± 41.9			4811.2 ± 24.3		150.1 ± 2.2		908.5 ± 21.3	306.0 ± 10.8	6671 ± 58.2
36	*M. sodiroana*	42.6 ± 0.1	196.7 ± 10.4	463.9 ± 18.8	5390.4 ± 28.8						73.5 ± 0.2	177.4 ± 3.0	101.5 ± 1.4	281.0 ± 13.3	6727.1 ± 328.6
37*	*G. ichthyoderma*	3.6 ± 0.1							3561.9 ± 79.8						6952.4 ± 153.6
38*	*H. episcopalis*	65.6 ± 0.9			59.3 ± 1.9	11.5 ± 0.9		185.5 ± 8.1			16.9 ± 1.0				338.9 ± 7.2
39	*H. collinsiana*	30.8 ± 1.4				52.6 ± 0.3					45.1 ± 1.7	73.0 ± 1.6	180.4 ± 0.6		431.0 ± 1.2
40	*H. latispatha*	36.1 ± 0.2			89.2 ± 7.2			125.8 ± 8.3		234.1 ± 2.6	18.5 ± 0.1	28.1 ± 0.6			531.8 ± 12.6
41	*H. rostrata*	44.7 ± 4.6		102.5 ± 6.4	387.5 ± 3.3						272.0 ± 17.3		403.6 ± 24.6	469.0 ± 6.2	1679.3 ± 49.6
42	*H. wagneriana*	359 ± 15.7		30.2 ± 1.3					432.3 ± 6.5		33.8 ± 1.6		140.6 ± 4.4	69.7 ± 1.6	1065.5 ± 6.0
43	*P. vulgaris*	18.3 ± 0.6					77.9 ± 1.2		771.8 ± 36.2	3784.4 ± 268.3	57.3 ± 0.6		80.9 ± 2.4		4790.5 ± 308.2
44*	*H. rosa-sinensis*	20.6 ± 0.3			7022.9 ± 330.0						263.8 ± 1.8		81.8 ± 1.0		8428.7 ± 334.9
45	*S. stromanthoides*	20.4 ± 1.4				11.0 ± 0.5	45.3 ± 2.4	214.9 ± 3.1	130.2 ± 7.2	636.7 ± 12.9	54.0 ± 0.1	66.6 ± 2.3	68.5 ± 1.7		1247.4 ± 9.9
46	*P. heteromallum*	54 ± 0.4		1850.7 ± 56.8		68.5 ± 2.3									1973.2 ± 54.1
47	*A. lepidotus*	32 ± 1.1	24.3 ± 0.8		313.4 ± 7.2			2799.6 ± 67.3							3169.3 ± 60.4
48*	*C. mollis*	172.8 ± 9.3		822 ± 13.4							724.4 ± 2.2		472.1 ± 5.7		2907.3 ± 19.2
49	*P. urvilleana*	97.9 ± 1.9		175.1 ± 7.5		480.8 ± 2.9					207.9 ± 10.3		229.2 ± 19.9		1190.8 ± 6.9
50	*M. velutina*	48.4 ± 0.3				20.0 ± 0.6		177.2 ± 11.0			90.5 ± 1.6				336.1 ± 12.9
51	*B. spectabilis*	429.7 ± 14.9			755.9 ± 10.9	92.6 ± 1.9			435.2 ± 19.5	2850.1 ± 66					4563.5 ± 40.6
52*	*A. comata*	57.4 ± 0.4						653.7 ± 20.4							5824 ± 68.2
53	*Oncidium sp.*	47.31 ± 1.2				262.2 ± 3.1		207.2 ± 17.8			110.7 ± 2.4		1071.4 ± 6.6	236.5 ± 6.3	1935.3 ± 37.4
54	*S. liliastrum*	68.5 ± 2.3		191.1 ± 0.2		345.9 ± 12.7	280.3 ± 3.8	163.1 ± 12.6			102.9 ± 4.1		354.3 ± 9.0		1506.1 ± 6.3
55	*M. erythrophylla*	5.0 ± 0.2	16.1 ± 0.3			226.7 ± 29.2	37.1 ± 3.3	702.4 ± 54.6	2959.5 ± 144.2	4814.2 ± 137.3	176.9 ± 17.3		383.1 ± 86.5	587.8 ± 68.5	110,363.7 ± 431.8
56	*M. philippica*						547.6 ± 28.2				1349.8 ± 32.6		940.8 ± 26.3	3552.1 ± 23.8	6390.3 ± 110.8
57	*P. angustifolia*	11.5 ± 1.6	231.4 ± 11.9			425.7 ± 7.3	173.3 ± 1.3		430.3 ± 3.0						1272.3 ± 19
58	*B. arborea*	316.3 ± 1.9						10,729.0 ± 167.0			91.1 ± 2.0		78.3 ± 2.8		11,214.7 ± 173.7
59	*I. gesnerioides*						245.8 ± 5.7		2738.4 ± 190.6		83.1 ± 2.7		55.1 ± 2.4		3122.4 ± 191.2
60	*B. americana*	95.5 ± 3.5						1279.4 ± 114.5			596.3 ± 0.0		825.9 ± 24.6		2797.1 ± 142.6
61	*B. versicolor*	106.2 ± 0.9			1231.2 ± 57.3			809.2 ± 30.9	2556.1 ± 293.8	775.9 ± 45.2	545.6 ± 42		203.3 ± 11.9		6227.5 ± 758.4
62	*S. dulcamara*			898.7 ± 2.4			3435 ± 65.4								4333.7 ± 67.8
63*	*L. rugulosa*	32.4 ± 0.8							2326.9 ± 1.6				2949.5 ± 27.9	998.0 ± 14.7	6368.6 ± 9.6
64*	*A. purpurata*	15.5 ± 0.6				558.3 ± 43.2		1410.9 ± 65.0			498.9 ± 19.0		235.6 ± 13.3	282.8 ± 11.8	3147.4 ± 172.4
65	*H. coronarium*	24.0 ± 0.6	4.6 ± 0.5				43.9 ± 0.8	112.9 ± 1.7	232.0 ± 2.9		56.9 ± 0.8		602.0 ± 1.4	88.6 ± 2.8	1164.8 ± 2.0
66	*H. coccineum*	13.3 ± 0.4				17.5 ± 0.4	43.1 ± 1.2	146.5 ± 17.0					41.8 ± 3.1	39.4 ± 0.7	
67	*R. alpinia*	6.3 ± 0.1													6.3 ± 0.2

Additional phenolics detected in species with *: 9*, catechin (468.3 ± 7.5 mg/100g); 12*, vanillic acid (7.3 ± 0.0 mg/100 g); 14*, synapinic acid (1431.8 ± 62.9 mg/100 g); 16*, vanillic acid (7.3 ± 0.4 mg/100 g); 22*, vanillic acid (25.8 ± 0.7 mg/100 g); 26*, vanillic acid (6.3 ± 0.7 mg/100 g) and catechin (23.0 ± 0.1 mg/100 g); 27*, vanillic acid (912.7 ± 30.9 mg/100 g); 29* catechin (34.9 ± 1.5 mg/100 g); 31*, vanillic acid (12.5 ± 0.0 mg/100 g). 37*, *o*-coumaric acid (3387.0 ± 73.9 mg/100 g); 38*, 49.0 ± 0.3 mg/100 g); 44*, naringenin (1039.7 ± 3.7 mg/100 g); 48*, myricetin (716.0 ± 6.9 mg/100 g); 52*, catechin (130.2 ± 9.1 mg/100 g) and naringenin (4982.7 ± 39.0 mg/100 g); 63*, vanillic acid (61.8 ± 1.2 mg/100 g); 64*, catechin (145.3 ± 20.7 mg/100 g).

**Table 5:** Average values of the zone of inhibition of the floral species were analyzed.

N°	Flower Extracts	Extract Concentration (mg/mL)	Zone of Inhibition (mm)					
			*Bacterial strain*				*Fungal strain*	
			*E. coli ATCC* 8739	*S. aureus ATCC* 6538P	*P. aeruginosa ATCC* 9027	*S. mutans* ATCC 25175	*C. albicans* ATCC 1031	*C. tropicalis* ATCC 13803
1	*M. erythrochlamys*	162.4	-	12.5 ± 2.1	-	-	-	-
2	*O. cuspidatum*	127.5	-	-	-	-	-	-
4	*S. oblonga*	157.8	-	11.5 ± 0.7	-		-	-
5	*T. grandiflora*	130.2	-	-	-	10.0 ± 0.0	-	-
6	*C. argentea*	104.4	-	11.5 ± 0.7	-	-	-	-
8	*A. cathartica*	66.7	-	7.5 ± 0.7	-	-	-	-
9	*P. fragrantissimum*	97.0	-	14.0 ± 0.0	10.5 ± 0.7	10.5 ± 0.7	-	-
10	*P. strictum*	127.9	10.5 ± 0.7	12.5 ± 0.7	-	10.0 ± 2.8	-	-
11	*X. robustum*	127.2	-	10.5 ± 0.7	10.0 ± 0.0	-	-	-
13	*D. pinnata*	132.7	-	8.5 ± 2.1		-	-	-
20	*A. corymbosa*	75.0	-	11.5 ± 0.7	-	-	-	-
21	*G. nicaraguensis*	128.6	-	-	-	-	-	-
22	*R. veitchii*	81.0	-	7.5 ± 0.7	-	-	-	-
26	*C. spiralis*	334.9	13.0 ± 0.0	11.0 ± 1.4	11.0 ± 0.0	-	-	-
30	*C. nobilis*	251.2	15.5 ± 0.7	20.5 ± 0.7	13.0 ± 2.8	16.0 ± 0.0	-	-
32	*Acalypha poiretii*	300.0	10.5 ± 0.7	17.0 ± 1.4	12.0 ± 4.2	13.0 ± 0.0	-	-
33	*B. macrophylla*	550.5	10.5 ± 0.7	16.0 ± 1.4	10.5 ± 0.7	17.0 ± 0.0	-	-
35	*E. americana*	223.1	10.5 ± 0.7	10.0 ± 0.0	-	15.5 ± 0.7	-	-
37	*G. ichthyoderma*	174.8	-	14.5 ± 0.7	-	-	-	-
38	*H. episcopalis*	255.6	-	11.5 ± 0.7	-	-	-	-
41	*H. rostrata*	281.2	-	13.5 ± 2.1	-	10.0 ± 0.0	-	-
42	*H. wagneriana*	71.9	-	7.0 ± 0.0	-	-	-	-
47	*S. stromanthoides*	336.9	-	10.0 ± 1.4	-	-	-	-
46	*P. heteromallum*	255.6	10.5 ± 0.7	18.5 ± 0.8	15.0 ± 1.4	21.0 ± 0.0	13.0 ± 0.0	-
50	*M. velutina*	159.2	-	12.0 ± 1.4	-	12.0 ± 0.0	-	-
51	*B. spectabilis*	143.3	-	13.0 ± 0.0	-	22.0 ± 0.0	-	-
52	*E. aurantiacus*	131.2	-	11.5 ± 0.7		-	-	-
53	*Oncidium sp*	110.1	-	-			-	-
55	*M. erythrophylla*	127.2	15.0 ± 0.0	14.5 ± 0.7			-	-
56	*M. philippica*	302.9	-	12.0 ± 1.4	-	-	-	-
57	*P. angustifolia*	125.6	-	8.5 ± 0.7	-	-	-	-
61	*B. versicolor*	132.5	9.0 ± 1.4	12.0 ± 0.0	-	-	-	-
62	*S. dulcamara*	111.3	-	-	-	-	-	-
65	*H. coronarium*	127.8	8.5 ± 0.7	15.0 ± 1.4	-	-	-	-
66	*H. coccineum*	146.2	-	8.0 ± 1.4	-	-	-	-
	Control *		21.0 ± 2.1	24.8 ± 1.8	24.0 ± 1.2	30.0 ± 3.9	12.9 ± 1.4	18.9 ± 3.0

Note: -, non-active at the tested concentration; *, Streptomycin for bacteria and fluconazole for fungi.

## Data Availability

The original contributions presented in this study are included in the article. Further inquiries can be directed to the corresponding author.
